# Janus electrocatalytic flow-through membrane enables highly selective singlet oxygen production

**DOI:** 10.1038/s41467-020-20071-w

**Published:** 2020-12-04

**Authors:** Yumeng Zhao, Meng Sun, Xiaoxiong Wang, Chi Wang, Dongwei Lu, Wen Ma, Sebastian A. Kube, Jun Ma, Menachem Elimelech

**Affiliations:** 1 State Key Laboratory of Urban Water Resource and Environment, Harbin Institute of Technology, Harbin, 150090 China; 2 Department of Chemical and Environmental Engineering, Yale University, New Haven, CT 06520-8286 USA; 3 School of Environment, Northeast Normal University, Changchun, 130024 China; 4 Department of Mechanical Engineering and Materials Science, Yale University, New Haven, CT 06511 USA

**Keywords:** Pollution remediation, Electrocatalysis, Chemical engineering

## Abstract

The importance of singlet oxygen (^1^O_2_) in the environmental and biomedical fields has motivated research for effective ^1^O_2_ production. Electrocatalytic processes hold great potential for highly-automated and scalable ^1^O_2_ synthesis, but they are energy- and chemical-intensive. Herein, we present a Janus electrocatalytic membrane realizing ultra-efficient ^1^O_2_ production (6.9 mmol per m^3^ of permeate) and very low energy consumption (13.3 Wh per m^3^ of permeate) via a fast, flow-through electro-filtration process without the addition of chemical precursors. We confirm that a superoxide-mediated chain reaction, initiated by electrocatalytic oxygen reduction on the cathodic membrane side and subsequently terminated by H_2_O_2_ oxidation on the anodic membrane side, is crucial for ^1^O_2_ generation. We further demonstrate that the high ^1^O_2_ production efficiency is mainly attributable to the enhanced mass and charge transfer imparted by nano- and micro-confinement effects within the porous membrane structure. Our findings highlight a new electro-filtration strategy and an innovative reactive membrane design for synthesizing ^1^O_2_ for a broad range of potential applications including environmental remediation.

## Abstract (web summary)

Electrocatalytic processes are promising for automated and scalable synthesis of singlet oxygen, but they are energy- and chemical-intensive. Here the authors present a Janus electrocatalytic membrane that selectively produces singlet oxygen with low energy consumption and free of chemical precursors.

## Introduction

The seminal work by H. Kautsky in 1938 revealed an exciting form of molecular oxygen, singlet oxygen (^1^O_2_), which later has been proven to be pivotal in the chemical and biomedical fields^1–3^. This unique nonradical derivative of oxygen, together with hydroxyl radicals (^•^OH), is considered the most potent among reactive oxygen species (ROS)^4,5^. Compared with the short-lived and unselective ^•^OH radical, the meta-stable ^1^O_2_, possessing an unoccupied π* orbital, shows high selectivity toward electron-rich substances^6,7^, such as pharmaceuticals^8^, unsaturated biomolecules^7,9^, and microbial pathogens^10^. Consequently, ^1^O_2_ targeted reactions are applied in diverse fields, including water decontamination^10^, photodynamic cancer therapy^11^, and green organic synthesis^12^.

Greater recognition of the paramount importance of ^1^O_2_ has motivated research for more effective ^1^O_2_ production. Current approaches for ^1^O_2_ production mainly include (i) photosensitization using elaborately designed photosensitizers (e.g., molecular dyes^13^ or quantum nanodots^14^) for visible or UVA light adsorption^8,15^ and (ii) enzymatic reactions (e.g., peroxidases^16^ and oxygenases^17^ catalysis) in biological systems, relying on rigorous pH and temperature conditions of the intracellular environment^7,18^. While both approaches face challenges for industrial scale-up, electrocatalysis (EC), a highly automated and facile technology, could offer more approachable ^1^O_2_ production pathways^19–21^. Specifically, EC enables the flexible generation of critical ^1^O_2_ precursors, such as hydrogen peroxide^22,23^ and hypochlorite^20,24^. In addition, as an alternative to photoexcitation or chemiexcitation, EC provides efficient electro-excitation to ^1^O_2_ precursors, such as cathodic activation of peroxymonosulfate^21^ and anodic excitation of ferrocene^25^.

EC, however, is a much less explored process for ^1^O_2_ generation compared with photosensitization or enzymatic reactions. Present EC pathways rely on high dosages of precursors, which compromise the environmental sustainability of EC^26,27^. Further, for effective oxidation of target molecules by the in situ generated ^1^O_2_, EC currently requires long reaction times (range of hours) and consequently consumes high electric energy^20,21^. Therefore, it is critical to improving EC efficacy by eliminating the use of chemicals, significantly shortening residence time, and enhancing the Faradaic efficiency of the process.

Porous membranes have emerged as ideal electrode substrates to enhance EC performance^28,29^. A variety of membrane-like structures have been proposed, including horizontally stacked lamellar channels^30^ and vertically aligned straight-through pores^31^. These porous substrates spatially confine reactants and electrified active sites at the micrometer- and nanometer-scale. Spatial confinement by membrane pores and channels allows for optimal utilization of electrons by reactants due to minimal diffusion distance and mass transfer enhancement^30,32^, thereby overcoming intensive chemical usage in conventional EC systems. In addition, studies also highlighted the importance of flow-through configuration of membrane electrodes, which renders electrons to flow parallel to the fluid flow direction, further enhancing the charge transfer rates compared to the conventional flow-by mode (i.e., when the flow direction and electric field direction are perpendiculars)^33,34^. To date, reported electrocatalytic membranes render only half-cell reaction (cathodic reduction or anodic oxidation) within a membrane substrate. In such cases, the electrode redox reactions are not fully utilized. Therefore, exploiting the electrocatalytic flow-through porous membrane configuration incorporating both cathodic reduction and anodic oxidation may offer an innovative strategy for ^1^O_2_ synthesis, featuring enhanced Faradaic and energy efficiency.

Herein, we conceive a Janus electrocatalytic membrane realizing ultra-efficient ^1^O_2_ production without the addition of chemical precursors. The Janus membrane (Pd–Pt–CM) features double-sided electrochemically reactive surfaces formed by sputtering palladium (Pd) and platinum (Pt) nanoparticles on the respective sides of a ceramic membrane (CM) substrate. We confirmed the co-existence of ^1^O_2_ and its precursors, and investigated their spatiotemporal distribution throughout the Pd–Pt–CM during electrocatalytic filtration using the Pd- and Pt-sides as cathode and anode, respectively. We then evaluated the efficacy of the in situ generated ^1^O_2_ for water decontamination using sulfamethoxazole as a model organic molecule in the electrocatalytic filtration process. We further proposed a mechanism for the stepwise electrocatalytic production of ^1^O_2_ by the Pd–Pt–CM through a set of sequential ROS-mediated redox chain reactions within distinct membrane regions. Overall, our findings demonstrate a new paradigm and electrochemical material platform for ^1^O_2_ production by using a Janus electrocatalytic flow-through membrane.

## Results

### Fabrication and properties of Pd–Pt–CM

**Fig. 1:**
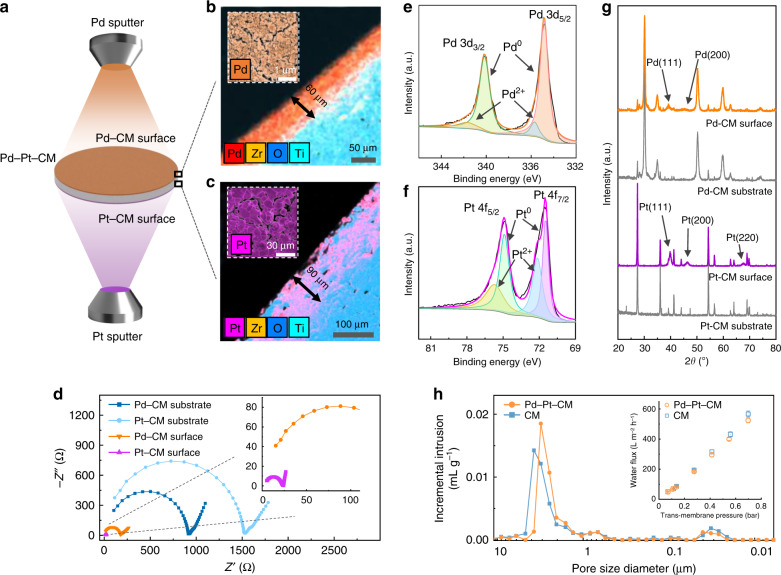
Fabrication and characterization of the Pd–Pt–CM. **a** Schematic depicting the fabrication of the Pd–Pt–CM by magnetron sputtering. **b**, **c** SEM cross-section images of the Pd–CM and Pt–CM surfaces, overlapped with EDS mapping of Zr (yellow), O (blue), Ti (green), and Pd (red) or Pt (purple) elements. The insets illustrate the SEM surface images of the respective functionalized membranes overlapped with EDS mapping of Pd (orange) or Pt (purple) element. **d** Nyquist plots of the Pd–CM and Pt–CM surfaces of the Pd–Pt–CM and corresponding CM substrates in 100 mM Na_2_SO_4_ solution at applied frequencies varied from 10^6^–1 Hz. **e**, **f** XPS spectra of the Pd *3d* and Pt *4f* on the Pd–Pt–CM, respectively. **g** Grazing incidence XRD patterns of the Pd–CM and Pt–CM surfaces of the Pd–Pt–CM with their respective CM substrates. **h** Pore size distributions of the Pd–Pt–CM (orange circles) and the pristine CM (blue squares). The inset shows the water fluxes of the Pd–Pt–CM and the pristine CM as a function of transmembrane pressure (error bars represent standard deviation from triplicate experiments).

Due to their intrinsic electrical insulating properties and large thickness^35–37^, CM can serve as ideal substrates for incorporating both cathodic and anodic electrodes on separate sides of CM. This unique feature allows for sequential electro-redox reactions occurring in different inner-porous CM regions during electrocatalytic filtration. As illustrated in Fig. 1a, the Pd–Pt–CM was synthesized via confocal magnetron co-sputtering (details in “Methods”). Palladium (Pd) and platinum (Pt) nanoparticles were, respectively, sputtered on the feed- and permeate-side surfaces of the CM (300 kDa, thickness of 2.5 mm). Noble metal nanoparticles are chosen as they exhibit high electrical conductivity and render efficient EC reactions with lasting stability^38,39^.

Discernible color transformations for both the Pd–CM and Pt–CM surfaces (Supplementary Fig. 1) confirmed the respective metal deposition. Figure 1b, c depicts the morphologies of the Pd–Pt–CM by scanning electron microscopy (SEM) overlapped with the elemental mapping of X-ray EDS. Sputtered Pd and Pt nanoparticles penetrated into the membrane surfaces (detailed EDS mapping results are shown in Supplementary Figs. 2 and 3), exhibiting notable sputtered depths of approximately 60 μm (Fig. 1b, orange) and 90 μm (Fig. 1c, purple), respectively. Such deep inner-pore sputtering guarantees EC reactions within the confined porous membrane structure, instead of only the membrane surface. The larger sputtered depth of Pt–CM than that of Pd–CM, under the same sputtering thickness, is attributed to the distinct asymmetric porous architecture of the pristine CM (Supplementary Fig. 4). In addition, Pd and Pt nanoparticles were uniformly deposited on the respective surfaces of the Pd–Pt–CM as shown by the even distribution of the EDS signals of Pd (Fig. 1b inset, orange) and Pt (Fig. 1c inset, purple), suggesting unimpeded electron transfer over the membrane surfaces during EC reactions.

Conductance properties of the Pd–Pt–CM were further quantified by electrochemical impedance spectroscopy (EIS). As illustrated in Fig. 1d, we observed a 6-fold and 40-fold decrease in the amplitudes of the semicircles for the Pd–CM and Pt–CM surfaces, respectively, compared to the corresponding sides of the pristine CM. This observation indicates that the sputtering of Pd and Pt nanoparticles notably reduces the charge transfer resistance and facilitates electron transfer of the functionalized membrane surfaces^40^. Further, the Pt–CM surface exhibits much lower charge transfer resistance than that of the Pd–CM surface, which could be attributed to the synergistic effect of the higher intrinsic conductivity of Pt and the larger sputtered depth.

The elemental composition and the crystalline structure of the Pd–Pt–CM were investigated by X-ray photoelectron spectroscopy (XPS) and the grazing incidence X-ray diffraction (GI-XRD). XPS peaks of Pd *3d*_3/2_ and Pd *3d*_5/2_, centered at 340.2 and 335.1 eV (Fig. 1e), suggest that Pd^0^ is the dominant component with minor Pd^2+^ coexisting on the Pd–CM surface^41^, possibly due to surface oxidation. Similarly, the deconvoluted peaks of Pt *4f*_5/2_ and Pt *4f*_7/2_ at binding energies of 74.8 and 71.5 eV (Fig. 1f) indicate the predominance of Pt^0^ along with partially oxidized Pt^2+^ on the Pt–CM surface^42^ (XPS survey spectra is provided in Supplementary Fig. 5). The Pt^2+^ signal is likely ascribed to the oxidation of Pt^0^ on the near-surface of the anodic membrane region since XPS can only retrieve information <10 nm past the surface of the sample^43^. Notably, the bulk sputtered Pt underneath the anodic membrane surface is mainly composed of Pt^0^, the more thermodynamically stable Pt species. The XRD analysis reveals that the Pd–CM surface (Fig. 1g, orange) exhibits characteristic diffraction peaks of the (111) and (200) planes of Pd^0^ (JCPDS No. 46-1043) with a face-centered cubic (fcc) crystal structure at 39.2° and 45.5°, respectively^44^. The other peaks correspond to rutile (JCPDS No. 73-1232) and zirconium oxide (JCPDS No. 49-1642), indicative of the CM substrate. The presence of crystalline Pt for the Pt–CM surface (Fig. 1g, purple) is corroborated by the diffraction peaks at 39.7°, 46.5°, and 67.7°, consistent with (111), (200), and (220) planes of the fcc Pt^0^ (JCPDS No. 04-0802)^45^. The mean nanoparticle sizes of Pd and Pt are calculated as 20.1 and 22.8 nm, respectively, using the Scherrer equation^46^. Notably, the high dispersion of the nano-sized Pd and Pt is indicated by the broadening of the XRD diffraction peaks^47^, as compared with the intensive peaks of the pristine CM grains (Fig. 1g, gray).

We further analyzed the influence of sputtering on membrane performance. The resembling pore size distributions (Fig. 1h) of the Pd–Pt–CM and the pristine CM suggest a negligible effect of sputtering on membrane structure. The inset in Fig. 1h also shows that the Pd–Pt–CM has comparable water flux as the pristine CM, indicating the negligible influence of sputtering on membrane permeability. We also observed reduced surface roughness of both functionalized surfaces of the Pd–Pt–CM comparing with those of the pristine CM (Supplementary Fig. 6), implying that the seeding of Pd and Pt nanoparticles smooths the surface morphology. In addition, the Pd–Pt–CM possesses hydrophilic surface properties as the pristine CM, as evidenced by the similar water contact angles of the Pd–Pt–CM (21°) and CM (19°) (Supplementary Fig. 7). The smooth surface and hydrophilic nature of the Pd–Pt–CM suggest a lessened fouling potential by organic molecules^35,37^ and thus more efficient electron transfer during filtration.

### Spatiotemporal distribution of ^1^O_2_ and other ROS in Pd–Pt–CM

**Fig. 2:**
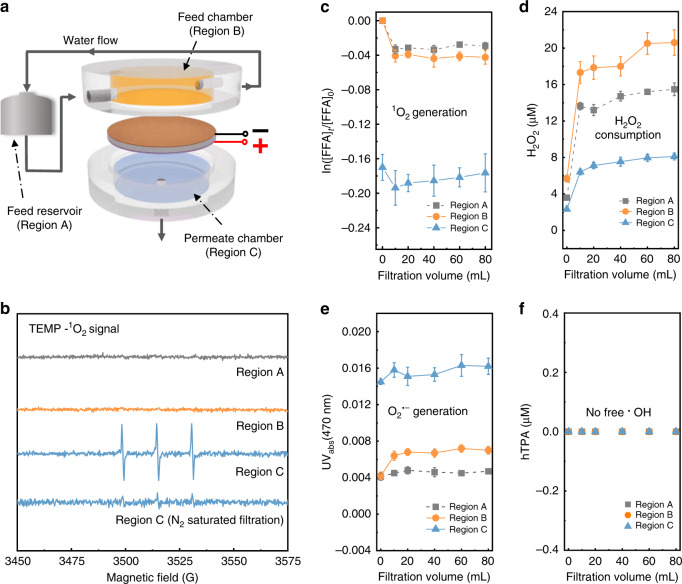
Spatiotemporal distribution of reactive oxygen species (ROS) generated in the Pd–Pt–CM. **a** Schematic illustrating the cross-flow filtration system containing a feed reservoir (Region A, gray) and an electrofiltration module with a 12 mL feed chamber (Region B, orange), a 12 mL permeate chamber (Region C, blue), and the Pd–Pt–CM. **b** EPR analysis for ^1^O_2_ in different regions of the filtration system using 2,2,6,6-tetramethylpiperidine (TEMP, 25 mM) as the trapping agent. EPR measurement of the TEMP-^1^O_2_ adduct in the permeate (Region C) of the filtration system using an N_2_-saturated feed solution is conducted for comparison. **c** ^1^O_2_ generation indicated by the degradation of furfuryl alcohol (FFA, 50 μM). **d** H_2_O_2_ detection measured by Amplex Red color reaction. **e** Detection of O_2_^**•**−^ by using 2,3-Bis-(2-methoxy-4-nitro-5-sulfophenyl)-2H-tetrazolium-5-carboxanilide (XTT, 100 μM) as the probe. **f** ^**•**^OH measurement by applying terephthalate (TPA, 1 mM) which reacts with ^**•**^OH to form hydroxyterephthalate (hTPA). Error bars represent standard deviations from triplicate experiments.

Electrocatalytic filtration with the Pd–Pt–CM was conducted in a customized cross-flow filtration device (Supplementary Fig. 8). As shown schematically in Fig. 2a, the Pd–Pt–CM was mounted in-between a feed chamber (Region B, orange) and a permeate chamber (Region C, blue). In our setup, the Pd–CM surface functioning as a cathode faced the feed chamber, while the Pt–CM surface serving as an anode faced the permeate. The feed solution was circulated within the feed reservoir (Region A, gray) at a cross-flow velocity of 0.8 L min^–1^ under an applied pressure of 0.1 bar.

We first detected singlet oxygen (^1^O_2_) generation by the Pd–Pt–CM electrofiltration with only Na_2_SO_4_ electrolyte as the feed solution. Electron paramagnetic resonance (EPR) spectra (Fig. 2b) showed that by applying the trapping agent 2,2,6,6-tetramethylpiperidine (TEMP), the typical triplet signal (1:1:1, a(N) = 16.9 G) of 2,2,6,6-tetramethyl-4-piperidinol-N-oxyl (TEMPO) in Region C (blue line) was observed, indicating the presence of ^1^O_2_ in the permeate^8,48^. Detailed EPR measurement is provided in the Supplementary methods; the EPR spectrum of the blank control solution, i.e., 100 mM Na_2_SO_4_ with 25 mM TEMP, is provided in Supplementary Fig. 9. The lack of TEMPO signals in Regions A and B (Fig. 2b, gray and orange lines) suggests that ^1^O_2_ was generated from the Pd–Pt–CM inner-pore structure during the electrocatalytic filtration. We observed a minimum applied voltage of 1.6 V for producing ^1^O_2_ (EPR spectra in Supplementary Fig. 10). Given that side reactions, i.e., water splitting for hydrogen and oxygen evolutions, are intensified at high voltage and thus reducing the Faradaic efficiency^49,50^, a minimum voltage of 1.6 V was selected for the subsequent experiments. In addition, ^1^O_2_ production was confirmed by furfuryl alcohol (FFA) probe^51^, which is highly selective to ^1^O_2_ with a rate constant of 1.2 × 10^8^ M^−1^ s^−1^. Near 17% degradation of FFA (50 μM) was continuously achieved by the Pd–Pt–CM electrified at 1.6 V (Fig. 2c, Region C, blue triangles), suggesting a constant production of ^1^O_2_ within the membrane. The detected products of FFA agree well with the oxidation products of FFA by ^1^O_2_ oxidation^32,52^ (Supplementary Fig. 11; detailed detection methods provided in the Supplementary methods). In contrast, less than 3% removal of FFA was observed from the Pd–CM surface (Fig. 2c, Region B, orange circles), likely due to adsorption of FFA on the Pd cathode^39^ (<1% FFA removal by conventional non-electrocatalytic Pd–Pt–CM filtration is shown in Supplementary Fig. 12, and <4.5% FFA removal by N_2_-saturated Pd–Pt–CM electrofiltration is shown in Supplementary Fig. 13).

Decreasing the oxygen level in the feed solution via nitrogen purging substantially reduced the triplet peak of TEMPO (Fig. 2b), suggesting oxygen to be the precursor for the electro-generated ^1^O_2_. Other ROS potentially produced in situ via the sequential electro-redox reaction during filtration, including hydrogen peroxide (H_2_O_2_), superoxide (O_2_^•−^), and hydroxyl radicals (^•^OH), could be involved as critical intermediates for ^1^O_2_ production^32,53^ (detailed ROS measurement provided in the Supplementary methods). Hydrogen peroxide (H_2_O_2_) was generated at the vicinity of the Pd–CM surface (Fig. 2d), which we attribute to the two-electron cathodic reduction of O_2_^50,54^. H_2_O_2_ tended to accumulate above the Pd–CM surface, as evidenced by the higher H_2_O_2_ concentration in Region B (Fig. 2d, orange circles) than that in Region A (Fig. 2d, gray squares). A near twofold decrease of H_2_O_2_ in the permeate (Fig. 2d, blue triangles) indicates that H_2_O_2_ was further consumed within the Pd–Pt–CM inner-pore structure. In contrast, superoxide (O_2_^•−^) was more notably produced within the Pd–Pt–CM, as evidenced by the higher concentration in the permeate (Fig. 2e, blue triangles) as compared with the other regions (Fig. 2e, orange circles and gray squares). O_2_^•−^ accumulation within the Pd–Pt–CM could be attributed to two pathways: (i) one-electron reduction of O_2_ via the Pd cathode^50^ and (ii) one-electron oxidation of H_2_O_2_ via the Pt anode^54,55^. In addition, free hydroxyl radicals (^•^OH) were not detected within the system (Fig. 2f); we attribute this observation to the intrinsic nature of Pt as an “active” anode, which exhibits low production of physisorbed Pt(^•^OH)^56^.

### Efficacy and energy efficiency of electrocatalytic ^1^O_2_ production

**Fig. 3:**
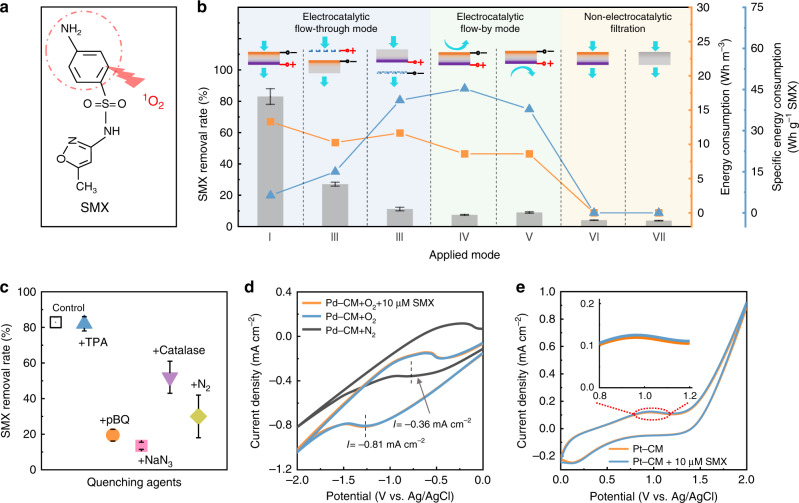
Effectiveness and energy efficiency of ^1^O_2_ in situ generated by the Pd–Pt–CM. **a** Schematic illustrating the reaction between ^1^O_2_ and SMX. **b** SMX removal efficiency and electric energy consumption by Pd–Pt–CM under different electrocatalytic and conventional filtration modes. Modes I–III are for flow-through electrofiltration. For mode, I, the Pd–CM and Pt–CM surfaces of the Pd–Pt–CM serve as cathode and anode, respectively. Modes II and III apply each side of the Pd–Pt–CM as the cathode (mode II) or anode (mode III) and a Ti mesh plate as the counter electrode inserted in the feed chamber (mode II) or permeate chamber (mode III). Modes IV and V are electrocatalytic flow-by modes using the Pd- and Pt-functionalized surfaces as the cathode and anode, respectively. The reaction times of modes IV and V are the same as the filtration duration in mode I (i.e., 38 min). Mode VI and VII are conventional filtration modes using Pd–Pt–CM and CM without applying voltage. For each filtration mode, the feed solution contains 10 μM SMX and 100 mM Na_2_SO_4_. Error bars represent the standard deviation from triplicate experiments. In the schematic above each bar, the orange layer and purple layer represent Pd–CM and Pt–CM surfaces, respectively, and the blue arrows denote the direction of water flow. **c** ROS quenching by applying 10 mM TPA, 10 mM p-benzoquinone (pBQ), 2 mg L^−1^ catalase, and 10 mM sodium azide (NaN_3_) as quenchers for ^**•**^OH, O_2_^**•**−^, H_2_O_2_, and ^1^O_2_, respectively. Measurements were performed in mode I with the respective quenching agent in the feed solution. Error bars represent the standard deviation from triplicate experiments. **d** CV curves of the Pd–CM surface in 100 mM Na_2_SO_4_ electrolyte at a scan rate of 0.1 V s^−1^. **e** CV curves of the Pt–CM surface in 100 mM Na_2_SO_4_ electrolyte at a scan rate of 0.1 V s^−1^.

The efficacy of the electrocatalytic Pd–Pt–CM for water decontamination was investigated. Sulfamethoxazole (SMX), an antibiotic molecule, was used as a model organic compound, which can be specifically attacked by ^1^O_2_ via the electron-rich aniline group^57^ (Fig. 3a). As shown in Fig. 3b, the Pd–Pt–CM (i.e., mode I) exhibited an ultra-efficient SMX removal (82.9%) during a single-pass electrofiltration (detailed SMX measurement provided in the Supplementary methods). The residence time within the membrane is ~23 s (Supplementary Eq. 3), which is two orders of magnitude lower than the reaction times in current flow-by EC configurations for water decontamination^21,58^. Notably, the uncompromised membrane permeability (Supplementary Fig. 14) indicates negligible fouling during the electrofiltration, further ensuring durable and unimpeded electron transfer within the membrane (as evidenced by the stable current curve in Supplementary Fig. 15). The electrocatalytic performance and stability of the Pd–Pt–CM were also well retained, as shown in Supplementary Fig. 16.

To validate the role of ^1^O_2_ and other ROS in the efficient SMX removal during the Pd–Pt–CM electrofiltration (i.e., mode I), we have conducted quenching tests using different ROS scavengers. It is well established that sodium azide (NaN_3_), p-benzoquinone (pBQ), catalase, and terephthalic acid (TPA) are specific scavengers for ^1^O_2_, O_2_^•−^, H_2_O_2_, and ^•^OH, respectively^7,59–61^. The efficient quenching using NaN_3_ confirms the main contribution of ^1^O_2_ to SMX removal in mode I (Fig. 3c). The oxidation products of SMX were also examined and were found to be consistent with oxidation products of SMX by ^1^O_2_ oxidation^57^ (Supplementary Fig. 17). Considering that ^1^O_2_ reacts with SMX at a minimal molar ratio of 1:1^57^, the minimum ^1^O_2_ yield by the Pd–Pt–CM can thus be calculated as 6.9 mmol per m^3^ of permeate, and the ^1^O_2_ yield rate is calculated as 0.3 μM s^−1^ accordingly (Supplementary Eqs. 4 and 5). In addition, given the considerably low reactivities of O_2_^•−^ and H_2_O_2_ with SMX (Supplementary Fig. 18), the observed quenching by pBQ and catalase suggests that O_2_^•−^ and H_2_O_2_ are critical intermediates to ^1^O_2_ production. The insignificant quenching by TPA indicates that free ^•^OH plays a negligible role in SMX removal in mode I. In addition, N_2_-saturated filtration with the Pd–Pt–CM significantly suppressed SMX removal, in agreement with the previous EPR result (Fig. 2b), indicating oxygen to be the source of the in situ generated ^1^O_2_.

We also investigated the contribution of direct electro-redox reactions on either side of the Pd–Pt–CM toward SMX removal via cyclic voltammetry (CV) measurements. At the cathodic Pd–CM surface (Fig. 3d), the distinctive CV peak (blue curve) and enhanced current density in the O_2_-saturated electrolyte compared with that in the N_2_-saturated electrolyte (black curve), corroborate the generation of H_2_O_2_ via oxygen reduction at the Pd–CM cathode. The addition of SMX to the Pd–CM cathode (Fig. 3d, orange curve) caused no obvious difference in CV curves, indicating inert interaction of SMX in direct electro-reduction. Similarly, for the anodic Pt–CM surface (Fig. 3e), the indiscernible difference in the CV curves following the addition of SMX suggests an insignificant influence of direct electro-oxidation towards SMX removal.

The efficacy of the Janus electrocatalytic Pd–Pt–CM (i.e., mode I) was further compared with those of other EC configurations, i.e., single-sided electrocatalytic flow-through Pd–Pt–CM (mode II and III) and electrocatalytic flow-by modes (mode IV and V) (detailed mode schematics in Supplementary Fig. 19). As we observe in Fig. 3b, mode I exhibited an over the threefold and sevenfold increase of SMX removal rate compared with mode II and mode III, respectively, highlighting the paramount importance of the Janus configuration of the electrocatalytic Pd–Pt–CM in enhancing SMX removal compared to single-sided electrocatalytic membrane configurations. Compared to mode I, mode IV and V demonstrated notably lower SMX removal rates of <10% by the Pd–Pt–CM, indicating an order of magnitude lower efficiency of flow-by mode than that of flow-through mode. Without adding electricity, conventional membrane filtration in modes VI and VII showed negligible SMX removal. In addition, following a similar trend as the SMX removal efficiency, mode I showed enhanced current density compared with modes II–V (Supplementary Fig. 15), which corroborates the improved transfer of electrons within the confined Janus Pd–Pt–CM porous structures. Taken together, these results collectively emphasize the key role of the electrocatalytic flow-through configuration using both sides of the Pd–Pt–CM as electrodes (i.e., mode I) for highly efficient ^1^O_2_ production and enhanced electron transfer in a single-pass filtration.

Electric energy consumption is another important consideration in the application of the electrocatalytic membrane. As observed in Fig. 3b (right axis, orange filled squares), mode I exhibited slightly higher energy consumption (detailed calculations in the Supplementary methods), i.e., 13.3 Wh m^−3^, than those in modes II–V (orange filled squares) mainly due to the higher current density. However, when taking the removal efficacy into account, the specific energy consumption of mode I (i.e., 6.33 Wh g^−1^ SMX) was significantly lower than those of modes II–V (Fig. 3b right axis, blue filled triangles), indicating an enhanced energy efficiency of the Janus electrocatalytic Pd–Pt–CM. We further compared the energy consumption of the Pd–Pt–CM (in mode I) with other reported electrocatalytic membranes for similar use of water decontamination (Supplementary Fig. 20). Remarkably, our Pd–Pt–CM (in mode I) had a substantially lower energy consumption than those reported in other studies.

## Discussion

**Fig. 4:**
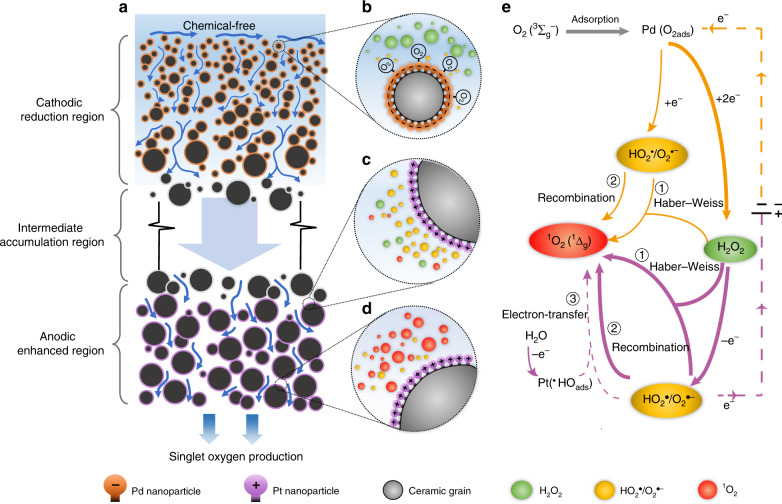
Proposed mechanism for ^1^O_2_ production by the electrocatalytic Pd–Pt–CM filtration. **a** Schematic describing the various regions in the Pd–Pt–CM. **b** Schematic of the cathodic Pd-functionalized (black and orange core–shell balls) membrane region, where H_2_O_2_ (green balls) and O_2_^**•**−^ (yellow balls) are generated via oxygen reduction in close vicinity to the Pd-functionalized pores. **c** Anodic Pt-functionalized (black and purple core–shell balls) membrane region, where H_2_O_2_ consumption, O_2_^**•**−^ accumulation, and ^1^O_2_ (red balls) generation occur concurrently. **d** A zoom-in close-to-bottom Pt-functionalized channel, where ^1^O_2_ is released throughout the permeate flow. **e** Proposed reaction pathway of singlet oxygen production. The orange and purple arrows represent the reactions occurring in the cathodic reduction region and anodic enhanced region, respectively. Dashed lines linked to the electric power supply denote the electron transfer.

Based on our findings discussed above, we propose the following mechanism for the in situ ^1^O_2_ production (Fig. 4). Due to the lack of TEMP-^1^O_2_ EPR fingerprint (Fig. 2b, blue lines) and the inhibited SMX removal (Fig. 3c, yellow diamond) in the absence of O_2_, we confirm that the presence of the ground state O_2_ is requisite for producing ^1^O_2_ by the Pd–Pt–CM. Differing from the general route of photosensitization, where sensitizers with the excited-triplet structures directly transfer energy from excitons to the ground state O_2_ for ^1^O_2_ generation^8,14^, the electrocatalytic Pd–Pt–CM does not serve as a medium for the direct activation from the inert O_2_ to ^1^O_2_. Alternatively, the Pd–Pt–CM allows indirect routes for ^1^O_2_ synthesis with the ROS H_2_O_2_ and O_2_^•−^ as critical intermediates, as confirmed by the inhibited SMX removal by quenching these ROS (Fig. 3c, orange circle, and purple triangle).

Figure 4a–d depict three functional regions in the porous Pd–Pt–CM undergoing a flow-through electrofiltration process (Fig. 4a) and the corresponding spatiotemporal distribution of ROS (Fig. 4b–d). H_2_O_2_ is first generated by an oxygen reduction reaction (ORR) in the cathodic Pd–CM region (Fig. 4b), followed by O_2_^•−^ accumulation chiefly by the subsequent oxidation of H_2_O_2_ in the anodic Pt–CM region (Fig. 4c)^50,54,55^. Benefiting from the continuous production of H_2_O_2_ and O_2_^•−^, the Pd–Pt–CM triggers two possible pathways for ^1^O_2_ formation: (i) the Haber–Weiss reaction between O_2_^**•**−^/HO_2_^**•**^ and H_2_O_2_ (Fig. 4e, route ①) and (ii) the recombination of O_2_^**•**−^/HO_2_^**•**^ (Fig. 4e, route ②)^32,53,62^. EPR tests further confirm the vital role of O_2_^•−^ and H_2_O_2_ in ^1^O_2_ formation (Supplementary Figs. 21 and 22). Although an ^•^OH radical-mediated route can also be feasible for ^1^O_2_ formation^32,53^ (Fig. 4e, route ③, dash line), this pathway is negligible due to the inhibition of the Pt anode for free ^•^OH generation, as Pt with its low oxygen evolution overpotential readily transforms the generated physisorbed Pt(^•^OH) into PtO superoxide^56^. This is evidenced by the absence of detected ^•^OH signal during electrofiltration (Fig. 2f) and the marginal inhibition of TPA (^•^OH scavenger) for SMX removal (Fig. 3c, blue triangle).

The formation of O_2_^•−^ intermediates by the H_2_O_2_ oxidation at the anodic Pt–CM region, as compared with that by the ORR pathway at the cathodic Pd–CM region, is critical for ^1^O_2_ synthesis in the Pd–Pt–CM (Fig. 4e, thick lines). As the Pd cathode favors the formation of H_2_O_2_ and H_2_O via a serial two-plus-two-electron transfer in ORR, its generation of O_2_^•−^ intermediates from the one-electron transfer is minor^63,64^. This proposed analysis is also verified by tuning the electrofiltration modes. By shifting from the use of only cathodic Pd–CM (Fig. 3b, mode II) to the use of sequential cathodic Pd–CM and anodic Pt–CM (Fig. 3b, mode I), a significant increase in the removal rate from 27.0 to 82.9% was observed, corroborating the key role of the anodic Pt–CM in contributing for O_2_^•−^ formation. Additionally, switching the electro-redox sequence, i.e., applying Pd–CM as anode facing the feed solution and Pt–CM as cathode facing the permeate, fails to produce ^1^O_2_ (Supplementary Figure 23). These results collectively validate that the electro-redox sequence, which induces sequential generation of H_2_O_2_ and O_2_^•−^, is critical for the ^1^O_2_ formation in the Pd–Pt–CM.

The distinct material structure of the Pd–Pt–CM, endowed by the confocal magnetron sputtering technique, offers additional mechanistic insights into the favorable ^1^O_2_ synthesis. One important structural feature achieved by the sputtering is the high dispersion of Pd and Pt nanoparticles (Fig. 1g), which prevents the formation of large agglomerates on the CM substrate^65^. This feature is also evidenced by the negligible changes in permeability and pore size distribution of the Pd–Pt–CM compared to the pristine CM (Fig. 1h). The high dispersion of Pd nanoparticles renders the favorable electrocatalytic formation of H_2_O_2_ over H_2_O in the ORR pathway, which is critical for inducing the subsequent ROS chain reactions (Fig. 4e, thick lines). Given the latter H_2_O product in ORR is formed by a desorption–readsorption of the H_2_O_2_ intermediate on a proximate Pd reactive site^64,66^, the increased interparticle distances of Pd nanoparticles imparted by sputtering prevent H_2_O_2_ from subsequent reduction by adjacent Pd agglomerates. Another important structural feature achieved by the Pd and Pt sputtering is the large sputtered depth in the near-surface regions of both sides of the Pd–Pt–CM (i.e., 60 and 90 μm for Pd–CM and Pt–CM regions from Fig. 1a, c). These sputtered depths are over an order of magnitude greater than the inner-porous reactive lengths of reported electrocatalytic membranes^67,68^. Taken together, the high dispersion of the electro-catalysts, as well as the elongated reactive regions, guarantee maximal exposure of the reactive sites in the Pd–Pt–CM. Furthermore, with the presence of the (100) facet of the Pt nanoparticles, the Pt anodic membrane region inherently favors the spin state of ^1^O_2_, thus likely facilitating ^1^O_2_ formation by promoting electron transfer between ^1^O_2_ precursors adsorbed on the Pt nanoparticles^61,69,70^ (for specific reaction steps see Supplementary Table 1).

Enhanced mass transfer induced by the flow-through electrofiltration is another important feature to promote in situ ^1^O_2_ formation. The forced convection, together with the confinement of the solution within the membrane inner-pores, synergistically enhances the transfer of electrons and reactants at the membrane-solution interface^33,71^. This synergy also facilitates the intimate contact of reactants to maximally exploit their redox capacity for ^1^O_2_ synthesis^72^. In addition, we carried out an SMX removal test in a batch mode without the forced filtration using the same electrocatalytic Pd–Pt–CM configuration as mode I. As shown in Supplementary Fig. 24, an over tenfold decrease of SMX removal efficiency (7.4%) and current density (0.006 mA cm^−2^) compared with those of mode I was observed, thus corroborating the enhanced mass and charge transfer by convection within the confined porous membrane regions.

In summary, we have developed a Janus electrocatalytic membrane, which leverages sequential reduction–oxidation reactions in distinct membrane regions for ultra-efficient ^1^O_2_ synthesis via a flow-through electrofiltration. This automated, sustainable and scalable approach of highly efficient ^1^O_2_ production and utilization has promising potential in diverse applications, including targeted water purification and in situ sensing in environmental remediation, green organic synthesis, and biomedical engineering. Moreover, we envision that the proposed Janus electrocatalytic membrane geometry, which is radically different from the general assembly of incorporating half-cell reaction with a membrane substrate, will pave the way for future smarter design strategies for high-performance and multifunctional electrocatalytic membranes.

## Methods

### Fabrication of Pd–Pt–CM

The Janus membrane (Pd–Pt–CM) was prepared via confocal magnetron co-sputtering (AJA International ATC 2200). Pd and Pt nanoparticles were sputtered on the feed- and permeate-side surfaces of a ceramic membrane (CM) substrate from respective targets with purity ≥99.95%. The sputtering targets were aligned in tetrahedral configuration with an angle and distance to the CM substrate set as 30° and 18 cm, respectively. A base pressure was retained at below 10^−7^ Pa prior to sputtering. Ultrapure argon gas was then employed for providing a working pressure of 0.3 Pa to eliminate possible contamination in the sputtering chamber. Subsequently, we placed the CM substrate on a silicon holder with its feed-side surface facing to the Pd target, followed by initiating Pd sputtering at a power of 30 W with a deposition rate of 2.1 nm min^−1^. The sputtering of Pt on the permeate-side surface of CM was then conducted at identical power with a deposition rate of 2.3 nm min^−1^. Deposition thicknesses for the Pd- and Pt-functionalized surfaces were controlled as ~30 nm by a quartz thickness gauge meter positioned in the center of the sputtering chamber.

### Characterization of Pd–Pt–CM

SEM (SU8230, Hitachi) coupled with X-ray energy dispersive spectroscopy (EDS, XFlash 5060FQ, Bruker) was employed to investigate the morphology and elemental distribution of the Pd–Pt–CM and the pristine CM substrate. The roughness of the Pd–CM surface and Pt–CM surface was measured by atomic force microscopy (Dimension Fastscan, Bruker) and a Zygo Nexview 3D optical profiler (AMETEK), respectively. Grazing incidence X-ray diffraction (GI-XRD, Smart lab, Rigaku) was applied for evaluating the crystal structures of the Pd–Pt–CM and the CM substrate. X-ray photoelectron spectroscopy (XPS, VersaProbe II, PHI) was used to characterize the elemental composition of the functionalized membrane surfaces. Membrane pore size distribution was measured by AutoPore V—mercury intrusion porosimetry. The water contact angle was measured by the sessile drop method using a contact angle goniometer (OneAttension, Biolin Scientific).

### Electrofiltration procedure

Electrofiltration experiments were conducted using a cross-flow membrane filtration system (Supplementary Fig. 8). The membrane system consists of a 12-mL feed chamber and a 12-mL permeate chamber. Pd–Pt–CM was inserted in-between the feed and permeate chambers; Pd–CM surface faced the feed chamber, while Pt–CM surface faced the permeate chamber. The Pd–CM and Pt–CM surfaces serving as cathode and anode, respectively, were connected to a DC supply (E3617A, HEWLETT PACKARD) via carbon tape. Porous titanium (Ti) mesh plate with an identical surface area as Pd–Pt–CM (17.34 cm^2^) was placed in each chamber and used as a counter electrode in specific flow-through modes (i.e., mode II and III in Fig. 3b). A constant voltage of 1.6 V was applied to the Pd–Pt–CM throughout the filtration experiments, and a multimeter (Fluke 87-V, Everett, WA) was used for current measurement.

Experiments were carried out in flow-through and flow-by modes. For each mode, feed solution was circulated between the feed chamber (i.e., Region B in Fig. 2a) and feed reservoir (i.e., Region A in Fig. 2a) at a flow velocity of 0.8 L min^−^^1^ by a gear pump (Cole-Parmer Instrument Company, Vernon Hills, IL, USA). In flow-through mode, the feed solution flowed through the membrane at a constant transmembrane pressure of 0.1 bar, while in the flow-by mode, the feed solution flowed tangent to the membrane surface.

### Electrochemical measurements

Electrochemical performance of the Pd–Pt–CM was analyzed by an electrochemical workstation (CHI 660E, CH Instruments) using a typical three-electrode configuration electrochemical cell. We applied one membrane surface as the working electrode, a platinum wire as the counter electrode, and an Ag/AgCl electrode as the reference electrode, in 100 mM Na_2_SO_4_ solution. EIS for each membrane surface was conducted by applying frequencies varied from 10^6^ to 1 Hz. CV curves of each membrane surface were collected at a scan rate of 0.1 V s^−1^ in either O_2_-saturated or N_2_-saturated Na_2_SO_4_ solution (100 mM). Pure nitrogen or oxygen gas was used to purge the electrolyte for 1 h prior to the tests.

## Supplementary information

Supplementary Information

## Data Availability

The data that support the findings of this study are available from the corresponding authors upon reasonable request.
