# Supplementary material for: Janus electrocatalytic flow-through membrane enables highly selective singlet oxygen production
Source: Nat Commun. 2020 Dec 4;11:6228. doi: 10.1038/s41467-020-20071-w (PMC7718259; doi:10.1038/s41467-020-20071-w)
Supplement: Supplementary file 1 — Supplementary Information [file 41467_2020_20071_MOESM1_ESM.pdf]

Supporting Information

Janus electrocatalytic flow-through membrane enables highly selective singlet oxygen production

Yumeng Zhao[1,2], Meng Sun[2]*, Xiaoxiong Wang[2], Chi Wang[2,3], Dongwei Lu[1], Wen Ma[2], Sebastian A. Kube[4], Jun Ma[1], and Menachem Elimelech[2]*

[1] State Key Laboratory of Urban Water Resource and Environment, Harbin Institute of Technology, Harbin 150090, China

[2] Department of Chemical and Environmental Engineering, Yale University, New Haven, Connecticut 06520-8286

[3] School of Environment, Northeast Normal University, Changchun 130024, China

[4] Department of Mechanical Engineering and Materials Science, Yale University, New Haven, Connecticut 06511

* Corresponding author:

E-mail: meng.sun@yale.edu;

E-mail: menachem.elimelech@yale.edu

Supplementary methods

Materials and chemicals

Sulfamethoxazole (SMX, Mw = 253.28 Da, ≥98%), 2,3-Bis-(2-methoxy-4-nitro-5-sulfophenyl)-2H-tetrazolium-5-carboxanilide (XTT, ≥90%), furfuryl alcohol (FFA, ≥97.5%), hydrogen peroxide (30% (w/w) in H_2O), Ampliflu Red (≥98.0%), terephthalic acid (TPA, ≥97.5%), p−benzoquinone (pBQ, ≥98.0%), horseradish peroxidase, catalase, 2,2,6,6-tetramethylpiperidine (TEMP, ≥99%), and sodium azide (NaN_3, ≥99.5%) were purchased from Sigma Aldrich (St. Louis, MO, USA). Sodium sulfate (Na_2SO_4) was purchased from J.T.Baker Chemicals (Phillipsburg, NJ, USA). Deionized (DI) water used for solution preparation and membrane rinsing was obtained from Milli−Q system (Millipore, Billerica, MA, USA). Ceramic membrane (CM) was purchased from Sterlitech (Kent, WA, USA) and immersed in DI water for 24 hours before use. Parameters of the CM are as follows: MWCO of 300 kDa, diameter of 47 mm, thickness of 2.5 mm and main components of TiO_2 and ZrO_2. More details in CM properties can be referred to the manufacturer.

Reactive oxygen species (ROS) and SMX measurement

ROS including $^1O_2$, $O_2^{\bullet-}$, $^{\bullet}OH$ and H_2O_2, were assessed in the Pd-Pt-CM electrocatalytic filtration using respective chemical probes. The electrocatalytic filtration procedure was as follows. Pd-CM and Pt-CM surfaces of the Pd-Pt-CM were connected to the cathode and anode of a power supply, respectively, at a voltage of 1.6 V. Pd-CM surface faced the feed chamber, while Pt-CM surface faced the permeate. A trans-membrane pressure of 0.1 bar and a cross-flow velocity of 0.8 L min^{-1} were applied during the filtration. Samples were taken from Region A, B and C (Figure 2a) at given filtration volumes for ROS detection.

$^1O_2$ generation was indicated by the degradation of selective probe FFA[1, 2], whose second-order rate constant with $^1O_2$ is reported to be 1.2×10^8 M^{-1} s^{-1}. 50 μM FFA was mixed with 100 mM Na_2SO_4 electrolyte as the feed solution. Aliquots of 200 μL from Region A, B and C (Figure 2a) at given electrocatalytic filtration volumes were inserted into the HPLC vial for measurement. The concentration of FFA was detected by Agilent high-performance liquid chromatography (HPLC) coupled with photodiode array detector (PDA; Agilent 1100). Sample of 50 μL was injected for each measurement at a temperature of 20 °C. Separation was conducted in an XDB-C18 column (5 μm, 4.6 × 150 mm, Agilent) with a mobile phase of 80% phosphoric acid (pH 2.3) and 20% acetonitrile (v/v) at a flow rate of 2 mL min^{-1}. FFA was quantified at adsorption wavelength of 220 nm and detected at a retention time of 1.35 min.

$O_2^{\bullet-}$ was examined by selective probe XTT[3, 4], which reacts with $O_2^{\bullet-}$ to generate XTT-

formazan. 100 μM XTT was mixed with 100 mM Na_2SO_4 electrolyte as the feed solution. Samples of 1 mL from Region A, B and C (Figure 2a) at given electrocatalytic filtration volumes were inserted into a cuvette. The detection of XTT−formazan was then conducted by UV−Vis spectrophotometer (RF-5301PC; Shimadzu) at wavelength of 470 nm.

H_2O_2 was detected by selective probe Amplifu Red[5, 6], which reacts with H_2O_2 in the presence of horseradish peroxidase forming resorufin. 100 mM Na_2SO_4 electrolyte was applied as the feed solution. Aliquots of 50 μL from Region A, B or C (Figure 2a) at given electrocatalytic filtration volumes were mixed with 50 μL Amplifu Red reagent, which contained Amplifu Red (100 μM), horseradish peroxidase (0.05 U mL^{-1}) and phosphate (50 mM at pH 7.4) buffer. Quantification of resorufin in the mixture solution was further conducted by Agilent HPLC coupled with PDA (Agilent 1100). Sample of 50 μL was injected for each measurement at a temperature of 20 °C. Separation was conducted in an XDB-C18 column (5 μm, 4.6 × 150 mm, Agilent) with a mobile phase of 55% sodium citrate and 45% methanol (v/v) at a flow rate of 2 mL min^{-1}, where resorufin was quantified at adsorption wavelength of 560 nm and detected at a retention time of 1.45 min.

$^{\bullet}OH$ was measured by selective probe TPA[7, 8], which reacts with $^{\bullet}OH$ forming hydroxyterephthalate (hTPA). 1 mM TPA was mixed with 100 mM Na_2SO_4 electrolyte as the feed solution. Aliquots of 200 μL from Region A, B and C (Figure 2a) at given electrocatalytic filtration volumes were inserted into the HPLC vial for measurement. The concentration of hTPA was detected by Agilent HPLC coupled with PDA (Agilent 1100). Sample of 50 μL was injected for each measurement at a temperature of 20 °C. Separation was conducted in an XDB-C18 column (5 μm, 4.6 × 150 mm, Agilent) with a mobile phase of 65% phosphoric acid (pH 2.3) and 35% methanol (v/v) at a flow rate of 2 mL min^{-1}, where hTPA was quantified at adsorption wavelength of 315 nm and detected at a retention time of 3.34 min.

SMX was detected by Agilent HPLC coupled with PDA (Agilent 1100). Sample of 50 μL was injected for each measurement at a temperature of 20 °C. Separation was conducted in an XDB-C18 column (5 μm, 4.6 × 150 mm, Agilent) with a mobile phase of 40% acetonitrile and 60% phosphoric acid (v/v) at a flow rate of 2 mL min^{-1}. SMX was quantified at adsorption wavelength of 277 nm and detected at a retention time of 1.83 min.

Electron paramagnetic resonance (EPR) measurement

Electron paramagnetic resonance (EPR) was conducted using a Bruker ELEXSYS E500 spectrometer equipped with an SHQ resonator. TEMP was applied as the trapping agent and can react with $^1O_2$ to form the 2,2,6,6-tetramethyl-4-piperidinol-N-oxyl (TEMPO) paramagnetic

adduct[9, 10]. During the electrocatalytic filtration, 25 mM TEMP was added into the 100mM Na_2SO_4 electrolyte as the feed solution. The electrocatalytic filtration procedure was as follows. Pd-CM and Pt-CM surfaces of the Pd-Pt-CM were connected to the cathode and anode of a power supply, respectively, at a voltage of 1.6 V. Pd-CM surface faced the feed chamber, while Pt-CM surface faced the permeate. A trans-membrane pressure of 0.1 bar and a cross-flow velocity of 0.8 L min^{-1} were applied during the filtration. The electrocatalytic filtration was conducted for 10 min, and samples were taken from different regions, i.e., Region A, B and C in Figure 2a, by a 100 μL capillary tube. Samples were then immediately inserted into the EPR cavity for measurement. The EPR spectra was measured at room temperature, with the instrument settings as follows: modulation frequency of 100 kHz, modulation amplitude of 1 G, microwave power of 0.2 mW, sweep width of 200 G, and sweep time of 167 s.

Liquid chromatography-mass spectrometry detection methods

A quadrupole time-of-flight liquid chromatography-mass spectrometry (QTOF LC-MS) system (Agilent 6545 series) was used to identify the oxidation products of SMX and FFA. The HPLC system was equipped with EclipsePlus C18 column (2.1×50 mm, 1.8 μm particle size) for separation. The mobile phase for the separation of FFA consists of Milli-Q water (A) and methanol (B), which varied linearly from 95/5 to 80/20 (v/v, A/B) from 0 min to 20 min; the flow rate was set as 0.2 mL min^{-1}. The mobile phase for the separation of SMX consists of Milli-Q water (A) and acetonitrile (C), which varied linearly from 20/80 to 95/5 (v/v, A/C) from 0 min to 10 min; the flow rate was set as 0.2 mL min^{-1}. In following MS analysis, ions were generated by an electrospray ion source (ESI). The MS analysis parameters for SMX were set as follows: positive mode (ESI^+); gas temperature, 320 ℃; drying gas, 8 L min^{-1}; capillary, 3000 V; nebulizer, 35 psi; skimmer voltage, 65 V; fragmentor 175 V. The MS analysis parameters for FFA were set as follows: negative mode (ESI^-); gas temperature, 320 ℃; drying gas, 8 L min^{-1}; capillary, 3000 V; nebulizer, 35 psi; skimmer voltage, 65 V; fragmentor 100 V. Oxidation products of FFA were analyzed by LC-MS; oxidation products of SMX were analyzed by LC-MS/MS.

Membrane permeability and residence time calculation

Membrane permeability (J, L m^{-2} h^{-1}) and the corresponding normalized permeate flux (NIF) are calculated as follows:

$$J = \frac{V}{A\Delta t} \quad (1)$$

$$NIF = J / J_0 \tag{2}$$

where V is the permeate volume (mL), A represents the membrane area (cm^2), Δt is the filtration time (min), and J_0 is the pure water flux ($L\ m^{-2}\ h^{-1}$).

Hydraulic residence time (*HRT*, s) within membrane is calculated as follows:

$$HRT = \frac{V_m}{AJ} \tag{3}$$

where V_m is the pore volume (mL) of Pd-Pt-CM measured by mercury intrusion porosimetry, A represents the membrane area (cm^2), and J ($L\ m^{-2}\ h^{-1}$) is the membrane permeability.

$^1O_2$ yield calculation

The minimum $^1O_2$ yield (Y) and yield rate (P) by the Pd-Pt-CM electro-filtration are calculated considering a minimal molar ratio of 1:1 between $^1O_2$ and reacted SMX[11]:

$$Y = R_1 - R_2 \tag{4}$$

$$\mathrm{P} = \frac{R_1 - R_2}{\mathrm{HRT}} \tag{5}$$

where R_1 is the amount of removed SMX ($\mu mol\ L^{-1}$) in mode I without the presence of ROS scavengers, R_2 is the amount of removed SMX ($\mu mol\ L^{-1}$) with the presence of NaN_3 ($^1O_2$ scavenger), and HRT is the hydraulic residence time within the membrane. (The respective SMX removal rates are illustrated in Fig. 3c.)

Energy consumption calculation

The energy consumption (*EC*, $Wh\ m^{-3}$) for the electrocatalytic removal of SMX is calculated by equation (6). Taking the removal efficiency into account, the specific energy consumption (*SEC*, $Wh\ g^{-1}SMX$) is calculated according to equation (7).

$$EC = \frac{UIt}{V} \tag{6}$$

$$SEC = \frac{EC}{RM} \tag{7}$$

where U is the applied voltage (V), I is the measured current (mA), t is the reaction time (min), V is the electrocatalytic filtration volume (mL), R is the amount of removed SMX ($\mu mol\ L^{-1}$), and M is the molecular weight of SMX ($253.3\ g\ mol^{-1}$).

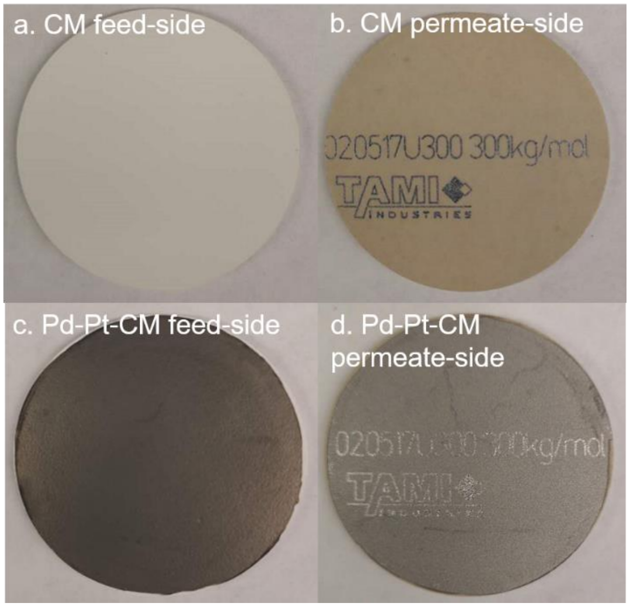

Supplementary Figure 1. Optical images of the Pd-Pt-CM and the pristine CM substrate: (a) feed side of CM, (b) permeate side of CM, (c) feed side of Pd-Pt-CM, and (d) permeate side of Pd-Pt-CM.

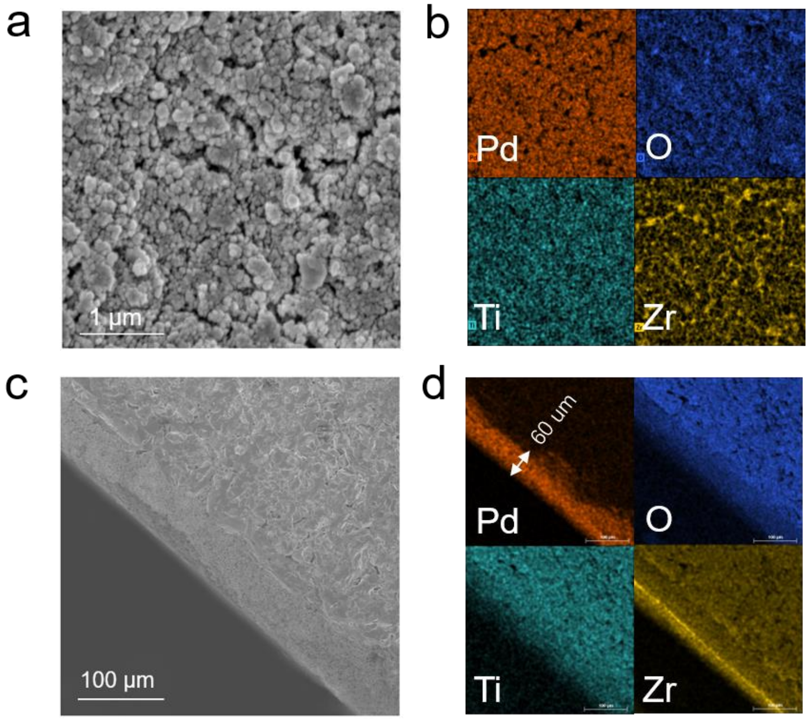

Supplementary Figure 2. SEM images (a and c) and corresponding EDS mapping (b and d) of Pd-CM surface and cross-section. Zr (yellow), O (blue), and Ti (green) elements are the main components of the pristine CM according to the manufacturer. The uniform signal of Pd (orange) element from the Pd-CM surface mapping (b) indicates the even distribution of sputtered Pd on membrane feed-side surface. The notable width of Pd (orange) signal from the Pd-CM cross-section mapping image (d) indicates a 60-μm intrusion depth of the sputtered Pd.

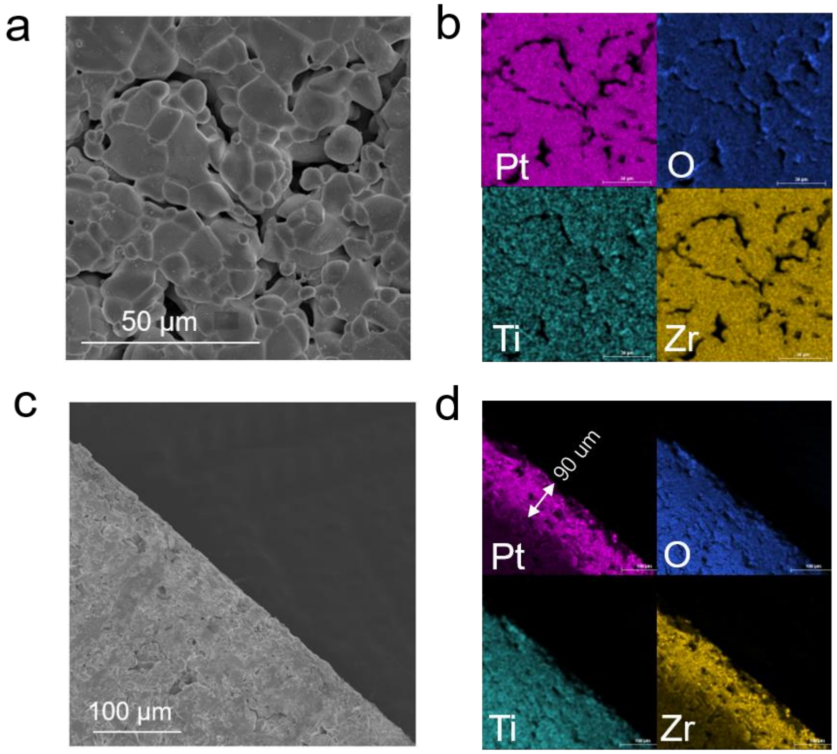

Supplementary Figure 3. SEM and EDS mapping images of Pt-CM surface (a and b) and corresponding cross-section (c and d). Pt (pink), Zr (yellow), O (blue), and Ti (green) elements are detected for respective elemental distribution. Homogeneous dispersion of Pt element from the Pt-CM surface suggests uniform Pt sputtering on the permeate-side of the CM substrate. Larger Pt-sputtered depth, i.e., 90 µm, on permeate-side of the CM substrate is noticed, as compared with the Pd-sputtered depth on feed-side of the membrane.

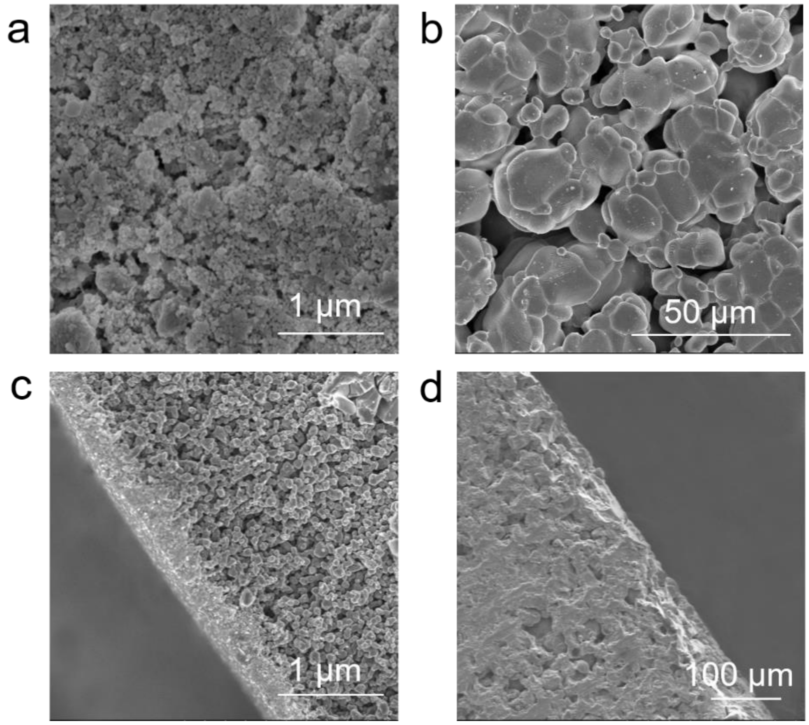

Supplementary Figure 4. SEM images of the pristine CM substrate, including feed-side surface (a) with corresponding cross-section (c), and permeate-side surface (b) with corresponding cross-section (d). Notable porous structures are detected from both surfaces, with the feed-side pores (a) considerably smaller than those of the permeate-side (b). Additionally, the cross-section images further exhibit the layered structure of the CM substrate, namely, dense porous structure from the feed-side followed by intermediate layer in-between (c) and subsequent loose macroporous structure from the permeate-side (d).

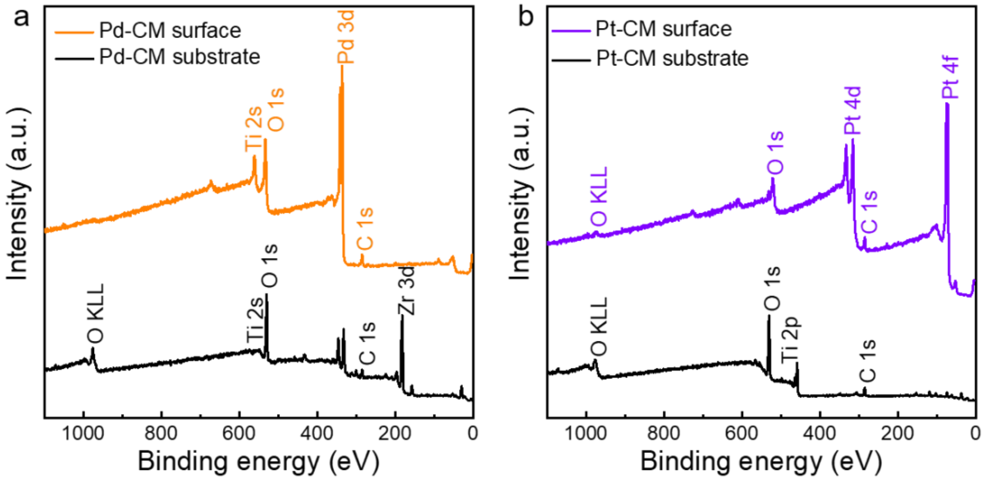

Supplementary Figure 5. XPS survey spectra of the Pd-Pt-CM and the CM substrate: (a) Pd-CM surface, (b) Pt-CM surface.

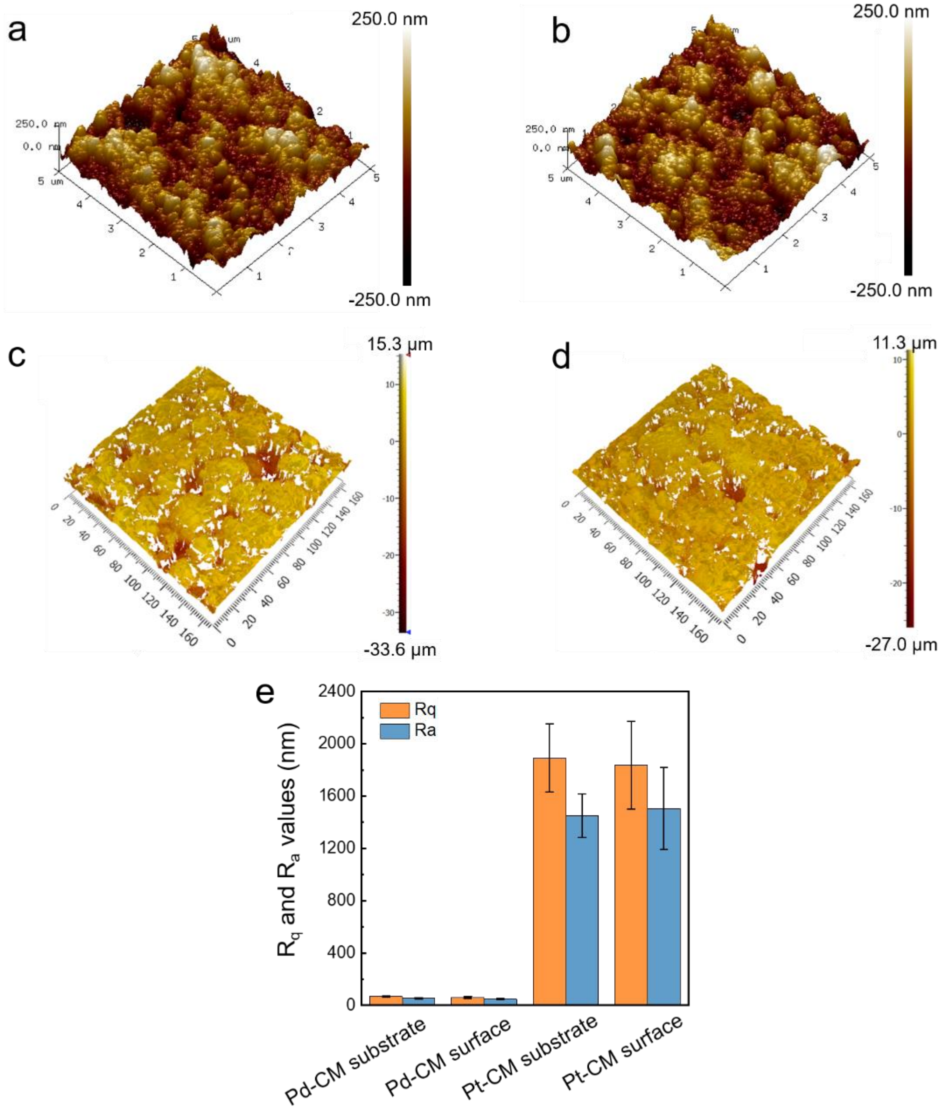

Supplementary Figure 6. Surface roughness of the Pd-Pt-CM and the CM substrate. (a) and (b) AFM 3D images of the Pd-CM surface and the corresponding CM substrate surface. (c) and (d) Zygo Nexview 3D images of the Pt-CM surface and the corresponding CM substrate surface. (e) Surface roughness parameters, i.e. mean square value (Rq) and average roughness (Ra), for both surfaces of the Pd-Pt-CM and CM. Each value is calculated from seven random locations of the membrane sample.

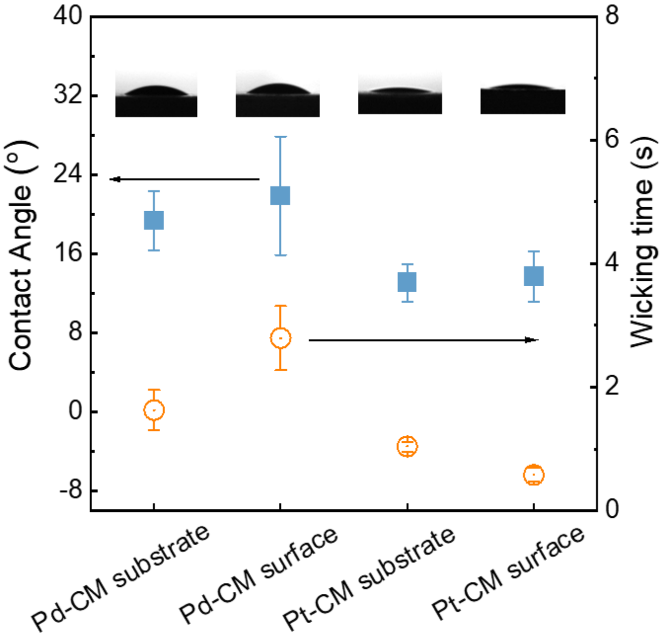

Supplementary Figure 7. Water contact angles (left Y-axis) of different membrane surfaces measured via the sessile drop method. The insets illustrate the state of a 3 µL-water droplet contacting the membrane surface at 0.34 s. As the Pd-Pt-CM and CM enabled water droplet to readily wet the membrane surfaces owing to the intrinsic hydrophilic properties and porous structures, the wicking times (from the time when water droplet contacts membrane surface till droplet total adsorption) were measured additionally (right Y-axis). Seven random locations from each membrane sample were selected for measurement. Similar water contact angles and wicking times for both surfaces of the Pd-Pt-CM and CM indicate resembling hydrophilic attributes of the membrane before and after metal sputtering.

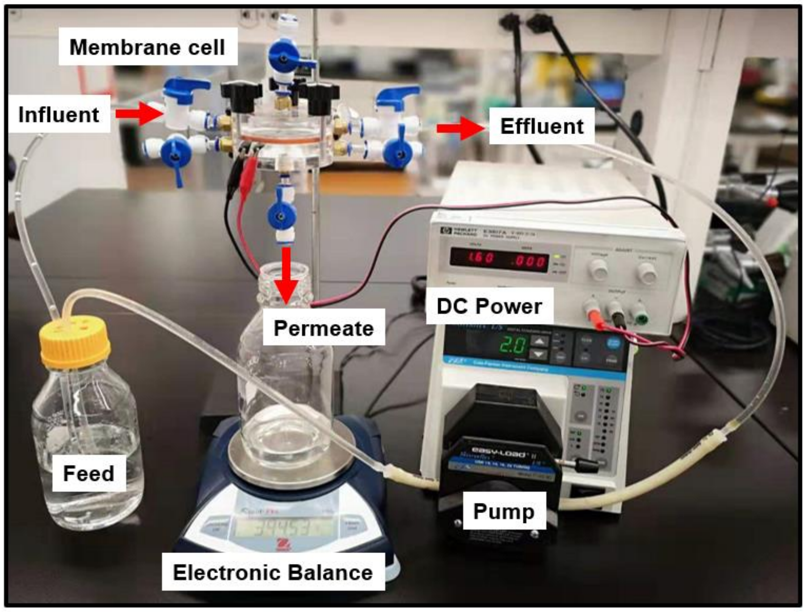

Supplementary Figure 8. Photograph of the experimental set-up, which contains the customized membrane cell, feed reservoir, permeate tank, DC power, gear pump, and electronic balance.

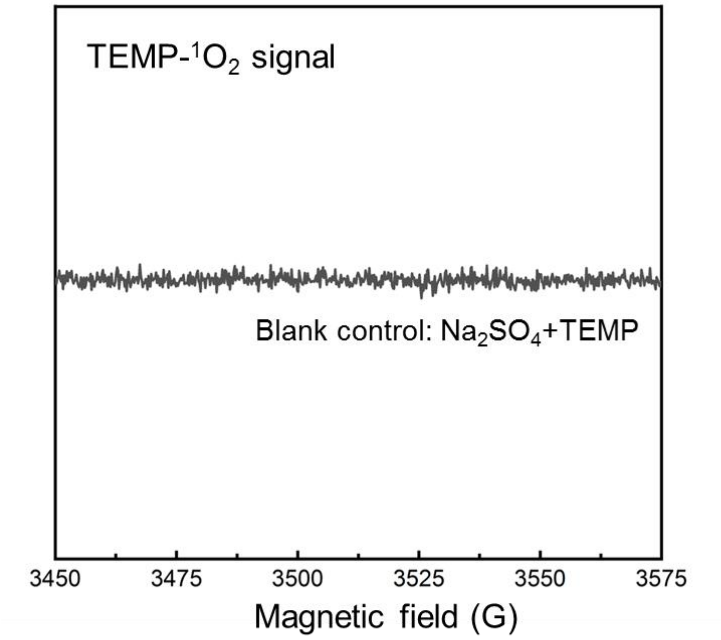

Supplementary Figure 9. EPR spectrum of the blank control solution (mixture of 100 mM Na_2SO_4 with 25 mM TEMP).

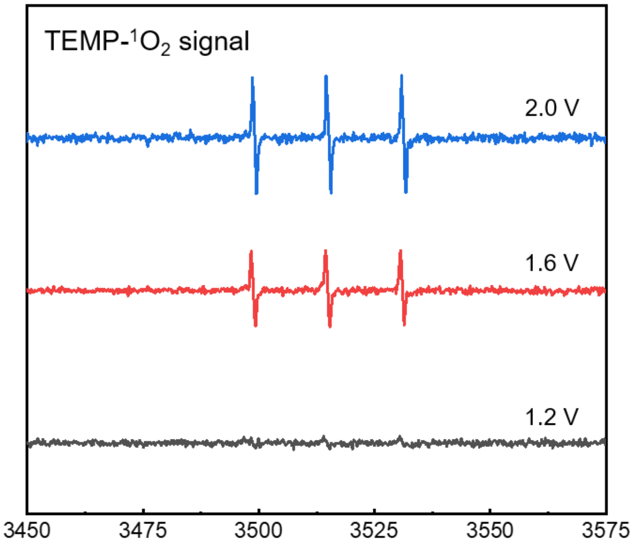

Supplementary Figure 10. EPR analysis for $^1O_2$ in Region C of the filtration system under different applied voltages. 2,2,6,6-tetramethylpiperidine (TEMP, 25 mM) was used as the trapping agent. The triplet TEMPO signal, indicative of the presence of $^1O_2$, appeared at a minimum voltage of 1.6 V.

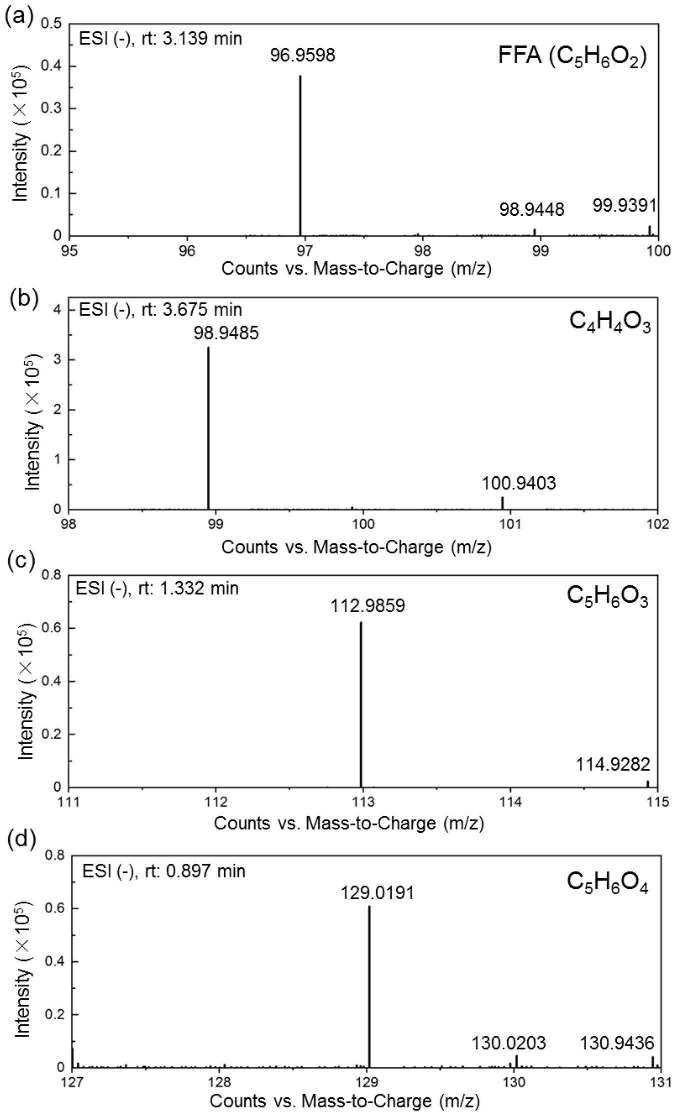

Supplementary Figure 11. Ion spectra of the detected (a) FFA, (b) $C_4H_4O_3$, (c) $C_5H_6O_3$, and (d) $C_5H_6O_4$ by LC-MS in negative ionization mode.

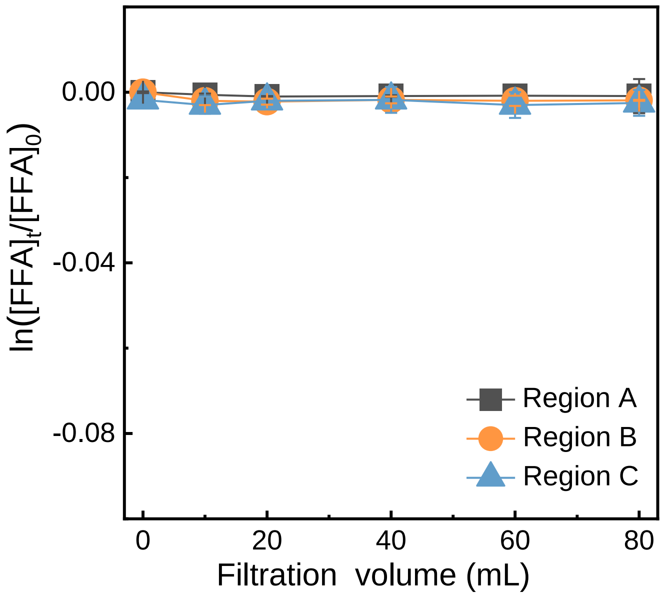

Supplementary Figure 12. Removal of furfuryl alcohol (FFA, initial concentration: 50 μM) under Pd-Pt-CM filtration without electricity. Filtration was conducted under the same condition as the electrocatalytic Pd-Pt-CM filtration with a trans-membrane pressure of 0.1 bar and a cross-flow velocity of 0.8 L min^{-1}. The feed solution also contained 100 mM Na_2SO_4 electrolyte. Error bars represent standard deviation from triplicate experiments.

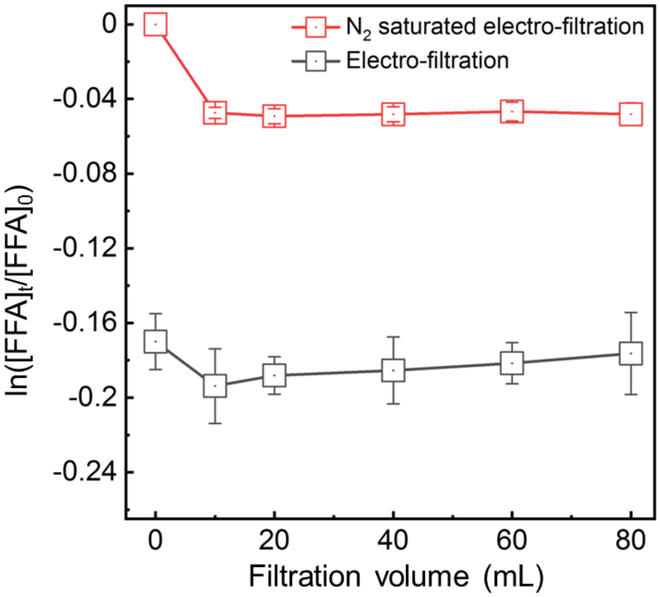

Supplementary Figure 13. Removal of FFA under N_2-saturated Pd-Pt-CM electro-filtration. The filtration conditions were as follows: applied voltage, 1.6 V; feed solution, 50 μM FFA with 100 mM Na_2SO_4 electrolyte; trans-membrane pressure, 0.1 bar; cross-flow velocity, 0.8 L min^{-1}; N_2 saturated filtration. FFA removal under Pd-Pt-CM electro-filtration (without purging N_2 gas, grey line) is also shown for reference. Error bars represent standard deviation from triplicate experiments.

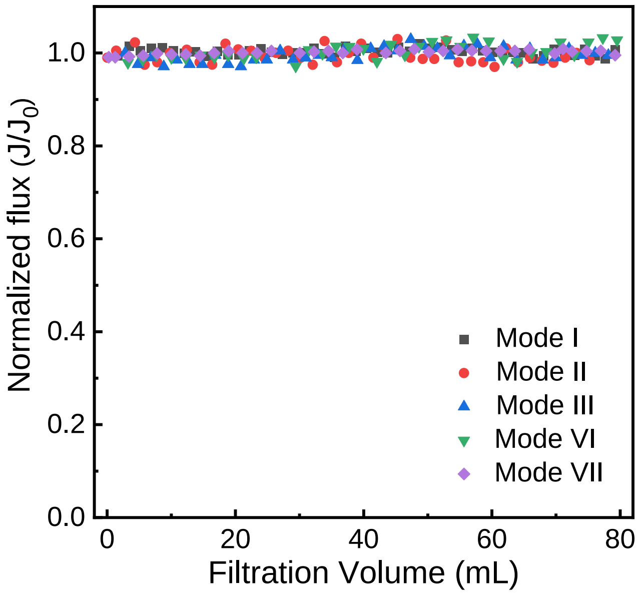

Supplementary Figure 14. Flux curves of mode I, II, III, VI and VII with feed solution containing 10 μM SMX and 100 mM Na_2SO_4. The horizontal flux curves indicate negligible fouling propensities during filtrations.

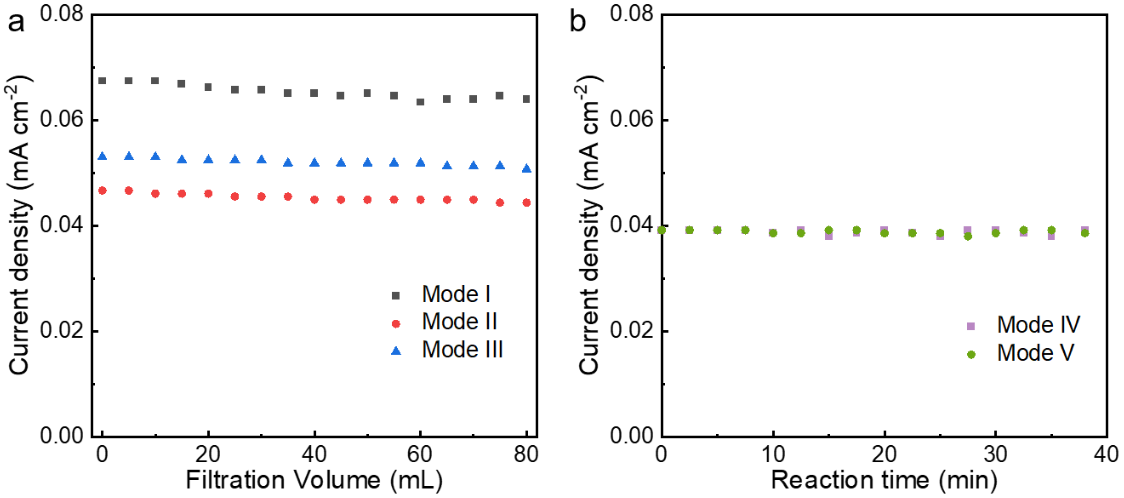

Supplementary Figure 15. Current curves of modes I-V with feed solution containing 10 μM SMX and 100 mM Na_2SO_4. The current values were collected from given filtrated volume intervals and given time intervals in modes I-III (a) and modes IV-V (b), respectively. The near horizontal current curves suggest the stable electrode performance of the electrocatalytic Pd-Pt-CM. The highest current density of Mode I indicates the enhanced mass transfer of electrons within the electrocatalytic flow-through Pd-Pt-CM.

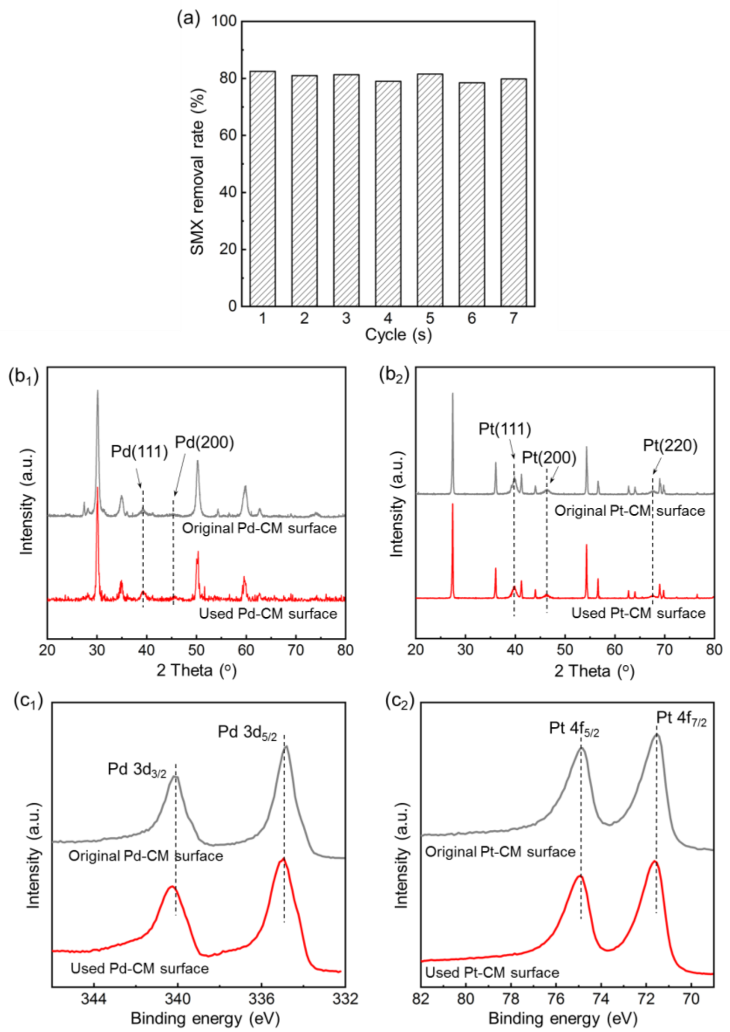

Supplementary Figure 16. Stability tests for the Pd-Pt-CM. (a) SMX removal performance by electrocatalytic Pd-Pt-CM filtrations for seven cycles. The filtration conditions for each cycle were as follows: applied voltage, 1.6 V; feed solution, 10 μM SMX with 100 mM Na_2SO_4 electrolyte; trans-membrane pressure, 0.1 bar; cross-flow velocity, 0.8 L min^{-1}; filtration volume, 80 mL. (b_1) XRD patterns for as-synthesized Pd-CM surface and used Pd-CM surface. (b_2) XRD patterns for as-synthesized Pt-CM surface and used Pt-CM surface. (c_1) XPS patterns for as-synthesized Pd-CM surface and used Pd-CM surface. (c_2) XPS patterns for as-synthesized Pt-CM surface and used Pt-CM surface.

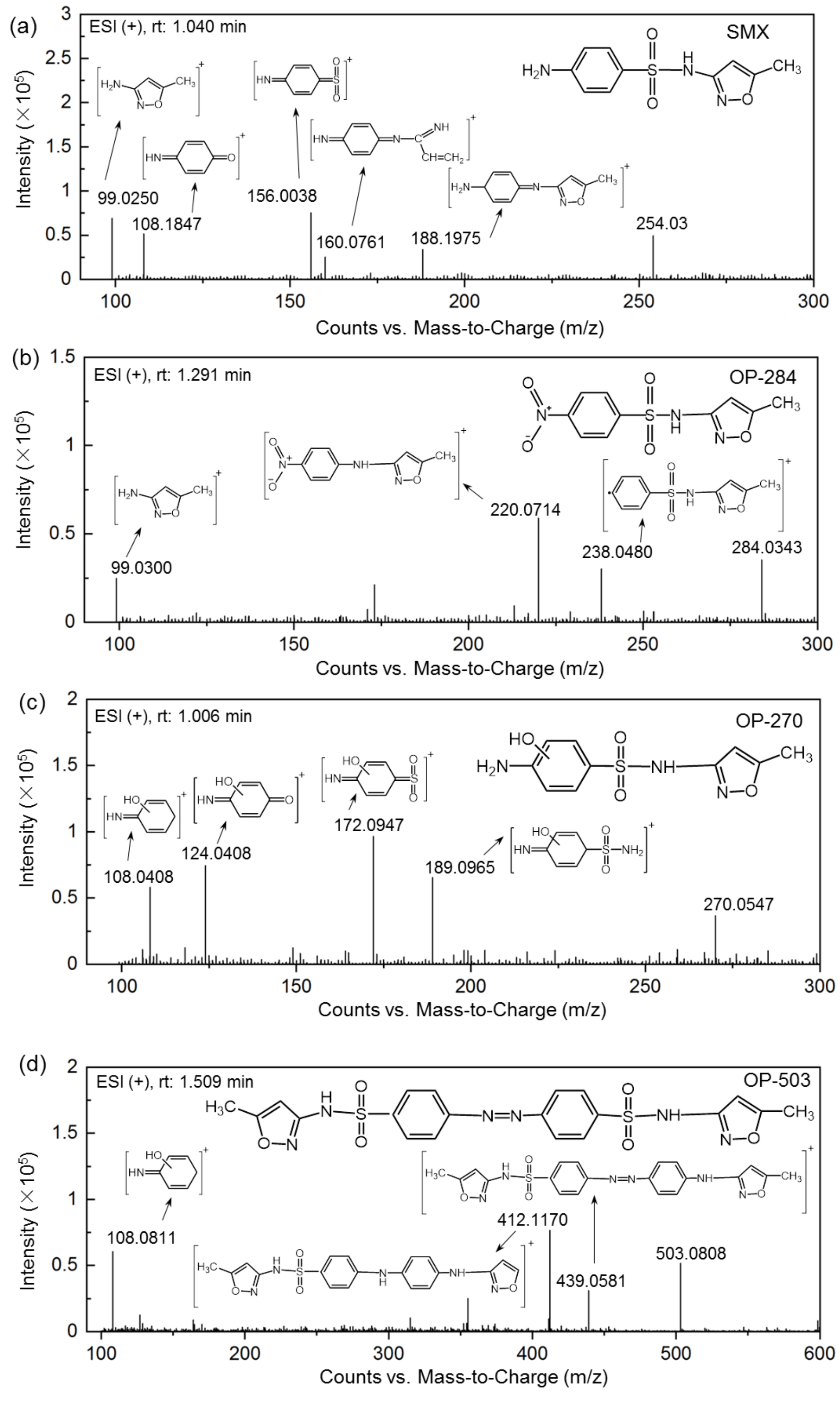

Supplementary Figure 17. Fragmented ion spectra of the detected (a) SMX, (b) OP-284, (c) OP-270, and (d) OP-503 by LC-MS/MS in positive ionization mode.

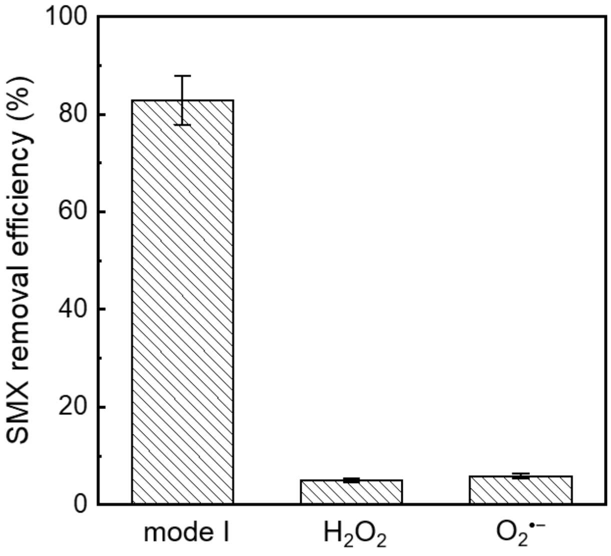

Supplementary Figure 18. SMX removal efficiency by H_2O_2 or $O_2^{\bullet-}$ during conventional CM filtration without the addition of electricity. The filtration condition was identical as mode I. 0.1 mM H_2O_2 was added into the feed solution for examining the influence of H_2O_2 to SMX removal. To investigate the influence of $O_2^{\bullet-}$, typical $O_2^{\bullet-}$ generator, i.e., mixed solution of 0.5 mM xanthine and 60 mU mL^{-1} xanthine oxidase (pH adjusted to 7.5 by phosphate buffer)[12], was added into the feed solution during filtration. The SMX removal efficiency of mode I is also depicted for comparison. Error bars represent standard deviation from triplicate experiments.

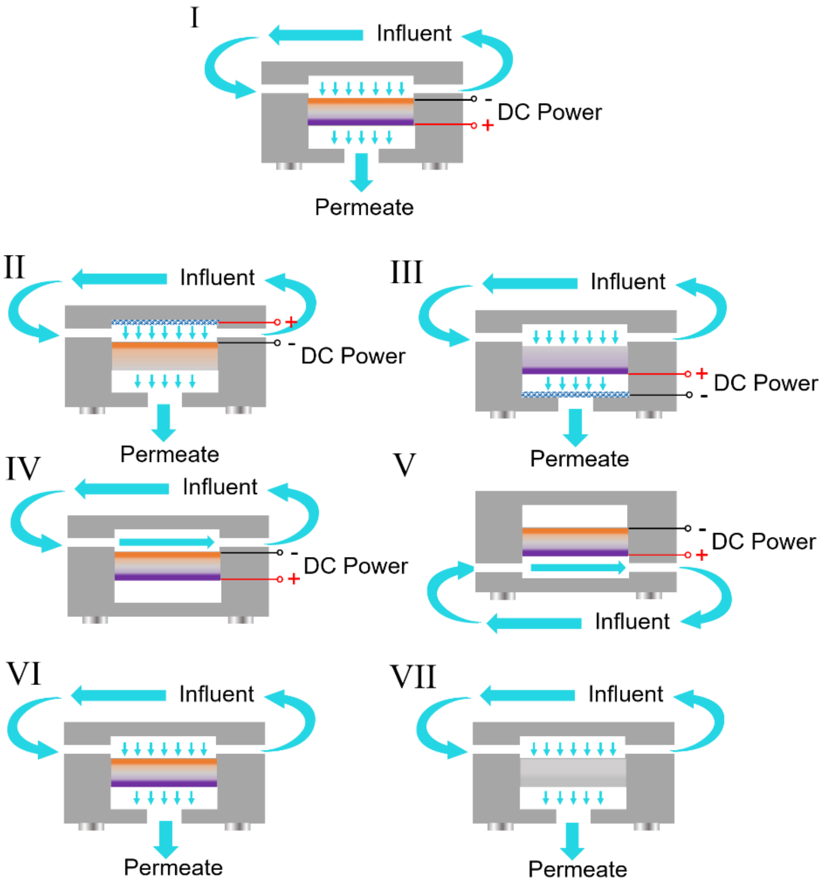

Supplementary Figure 19. Schematics of the applied seven filtration modes. For mode I, the Pd- and Pt-functionalized surfaces on the Pd-Pt-CM are used as cathode and anode, respectively. Mode II and III apply each one side of the Pd-Pt-CM as the cathode (i.e., mode II) or anode (i.e., mode III) with the porous Ti mesh plate as the counter electrode. Modes IV and V are electrocatalytic flow-by modes using the Pd- and Pt- functionalized surfaces as the cathode and anode, respectively. Feed solution is flown by Pd-CM surface in Mode IV or Pt-CM surface in Mode V. Modes VI and VII are conventional non-electrocatalytic filtrations using the Pd-Pt-CM and the CM substrate.

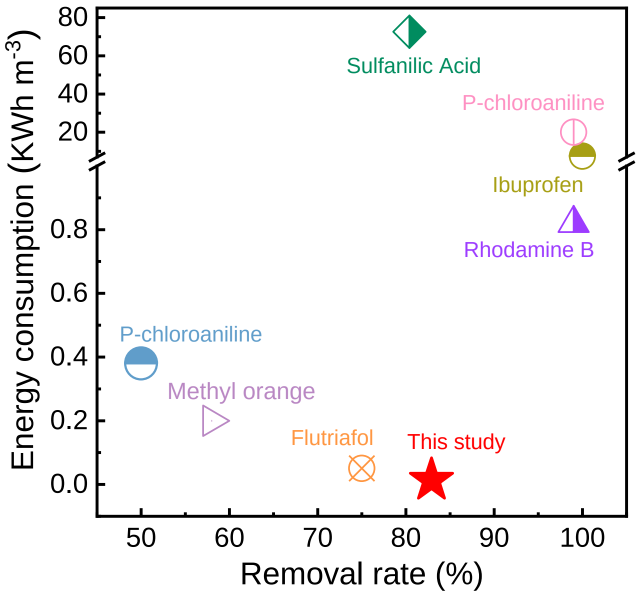

Supplementary Figure 20. Comparison of electrocatalytic membranes in various research studies[13, 14, 15, 16, 17, 18, 19] regarding the electric energy consumptions and corresponding micropollutant removal rates. The energy consumption and removal rate of mode I in this study are also indicated in the graph.

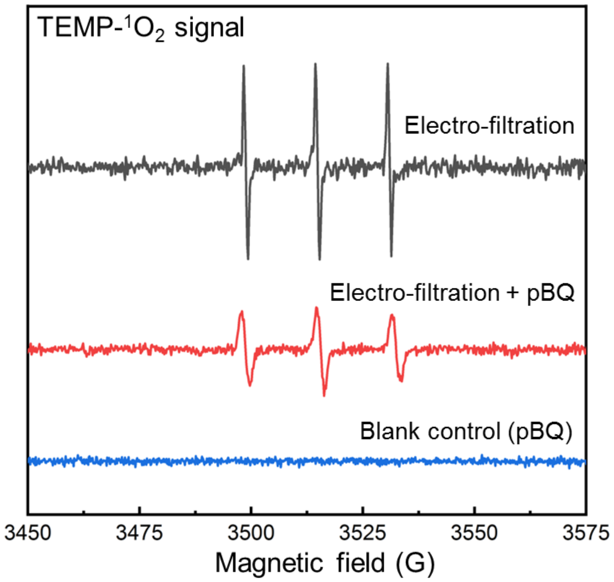

Supplementary Figure 21. Influence of $O_2^{\bullet-}$ quenching by pBQ (100 mM) on $^1O_2$ production in Region C (i.e., permeate) of the Pd-Pt-CM electro-filtration system. The filtration condition was identical as described in Fig. 2a. 2,2,6,6-tetramethylpiperidine (TEMP, 25 mM) was used as the trapping agent for the EPR analysis. The notable quenching effect by the addition of pBQ suggests $O_2^{\bullet-}$ as intermediate for $^1O_2$ production.

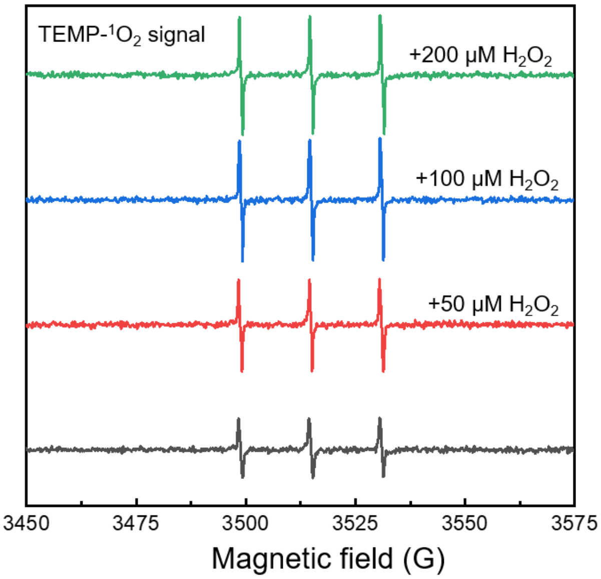

Supplementary Figure 22. Influence of H_2O_2 addition on $^1O_2$ production in Region C (i.e., permeate) of the Janus electrocatalytic Pd-Pt-CM filtration system. The filtration condition was identical as described in Fig. 2a. 2,2,6,6-tetramethylpiperidine (TEMP, 25 mM) was used as the trapping agent for the EPR analysis. The enhanced TEMP-$^1O_2$ EPR signal by the addition of H_2O_2 corroborates the involvement of H_2O_2 for $^1O_2$ production.

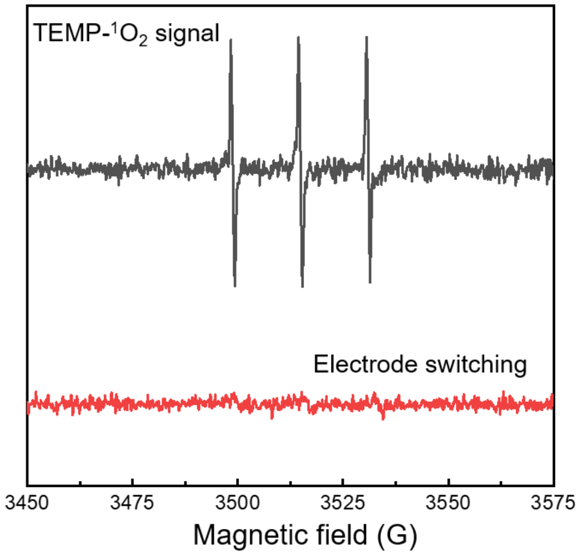

Supplementary Figure 23. Influence of electrode switching on $^1O_2$ production in Region C (i.e., permeate) of the Janus electrocatalytic Pd-Pt-CM filtration system. 2,2,6,6-tetramethylpiperidine (TEMP, 25 mM) was used as the trapping agent for the EPR analysis. The filtration condition was as follows: trans-membrane pressure of 0.1 bar, cross-flow velocity of 0.8 L min^{-1}, and feed solution containing 100 mM Na_2SO_4 and 25 mM TEMP. During the filtration, Pd-CM surface faced the feed solution, while Pt-CM surface faced the permeate. The black line indicates that Pd-CM and Pt-CM surfaces of the Pd-Pt-CM served as the cathode and anode, respectively, at a voltage of 1.6 V. On the contrary, the red line indicates that Pd-CM and Pt-CM surfaces of the Pd-Pt-CM served as the anode and cathode, respectively, at the same voltage of 1.6 V.

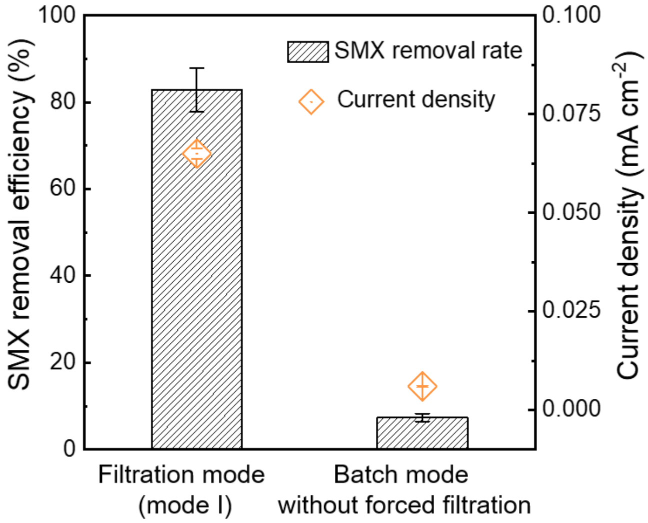

Supplementary Figure 24. Influence of the forced filtration on the SMX removal efficiency and current density of the electrocatalytic Pd-Pt-CM. Mode I was carried out under forced filtration, as described in Fig. 3b. The batch mode was carried out in a 100 mL beaker with magnetic stirring at 500 rpm. Feed solution identical as that in mode I (i.e., 80 mL mixed solution of 10 μM SMX and 100 mM Na_2SO_4) was prepared in the beaker. Pd-Pt-CM was then loaded into the solution to activate the reaction. Same as mode I, Pd-CM and Pt-CM served as cathode and anode, respectively, under the applied voltage of 1.6 V, and the reaction time of the batch mode is the same as the filtration duration in mode I. The values of the current density are derived from the average current values collected from given filtrated volume intervals and given time intervals in mode I and batch mode, respectively. Error bars represent standard deviation from triplicate experiments.

Supplementary Table 1. $^1O_2$ formation in the anodic Pt-CM region

$2O_2{}^{\bullet -}{}_{ads} + 2H^+ \xrightarrow{Pt(100)} {}^1O_2 + H_2O_2$	(1)
$O_2{}^{\bullet -}{}_{ads} + H_2O_2{}_{ads} \xrightarrow{Pt(100)} HOOOH_{intermediate} \rightarrow {}^1O_2 + H_2O$	(2)[20,21]

Supplementary References:

1. Appiani E, Ossola R, Latch DE, Erickson PR, McNeill K. Aqueous singlet oxygen reaction kinetics of furfuryl alcohol: effect of temperature, pH, and salt content. *Environ Sci Process Impacts.* **19**, 507-516 (2017).
2. Bokare AD, Choi W. Singlet-oxygen generation in alkaline periodate solution. *Environ. Sci. Technol.* **49**, 14392-14400 (2015).
3. Azócar MI, Alarcón R, Castillo A, Blamey JM, Walter M, Paez M. Capping of silver nanoparticles by anti-inflammatory ligands: antibacterial activity and superoxide anion generation. *J. Photochem. Photobiol. B, Biol.* **193**, 100-108 (2019).
4. Kim C, Park H-j, Cha S, Yoon J. Facile detection of photogenerated reactive oxygen species in TiO2 nanoparticles suspension using colorimetric probe-assisted spectrometric method. *Chemosphere.* **93**, 2011-2015 (2013).
5. Chu C*, et al.* Photochemical and nonphotochemical transformations of cysteine with dissolved organic matter. *Environ. Sci. Technol.* **50**, 6363-6373 (2016).
6. Lu X*, et al.* Tuning the permselectivity of polymeric desalination membranes via control of polymer crystallite size. *Nat. Commun.* **10**, 1-7 (2019).
7. Page SE, Arnold WA, McNeill K. Terephthalate as a probe for photochemically generated hydroxyl radical. *J. Environ. Monit.* **12**, 1658-1665 (2010).
8. Sun M*, et al.* Reinventing Fenton chemistry: Iron oxychloride nanosheet for pH-insensitive H2O2 activation. *Environ. Sci. Technol. Lett.* **5**, 186-191 (2018).
9. Rengifo-Herrera J, Pierzchała K, Sienkiewicz A, Forró L, Kiwi J, Pulgarin C. Abatement of organics and Escherichia coli by N, S co-doped TiO2 under UV and visible light. Implications of the formation of singlet oxygen (1O2) under visible light. *Appl. Catal. B.* **88**, 398-406 (2009).
10. Yang Y, Banerjee G, Brudvig GW, Kim J-H, Pignatello JJ. Oxidation of organic compounds in water by unactivated peroxymonosulfate. *Environ. Sci. Technol.* **52**, 5911-5919 (2018).
11. Zhou Y*, et al.* Activation of peroxymonosulfate by phenols: Important role of quinone intermediates and involvement of singlet oxygen. *Water Res.* **125**, 209-218 (2017).
12. Gyllenhammar H. Lucigenin chemiluminescence in the assessment of neutrophil superoxide production. *J. Immunol. Methods.* **97**, 209-213 (1987).
13. Bakr AR, Rahaman MS. Crossflow electrochemical filtration for elimination of ibuprofen and bisphenol a from pure and competing electrolytic solution conditions. *J. Hazard. Mater.* **365**, 615-621 (2019).
14. Li X*, et al.* Using TiO2 mesoflower interlayer in tubular porous titanium membranes for enhanced electrocatalytic filtration. *Electrochim. Acta.* **218**, 318-324 (2016).
15. Liu S*, et al.* Improved degradation of the aqueous flutriafol using a nanostructure macroporous PbO2 as reactive electrochemical membrane. *Electrochim. Acta.* **253**, 357-367 (2017).
16. Yang K*, et al.* A reactive electrochemical filter system with an excellent penetration flux porous Ti/SnO 2–Sb filter for efficient contaminant removal from water. *RSC Adv.* **8**, 13933-13944 (2018).
17. Zheng J, Wang Z, Ma J, Xu S, Wu Z. Development of an electrochemical ceramic membrane filtration system for efficient contaminant removal from waters. *Environ. Sci. Technol.* **52**, 4117-4126 (2018).
18. Zheng J, Xu S, Wu Z, Wang Z. Removal of p-chloroaniline from polluted waters using a cathodic electrochemical ceramic membrane reactor. *Sep. Purif. Technol.* **211**, 753-763 (2019).
19. Zheng J, Yan K, Wu Z, Liu M, Wang Z. Effective Removal of Sulfanilic Acid From Water Using a Low-Pressure Electrochemical RuO2-TiO2@ Ti/PVDF Composite Membrane. *Front. Chem.* **6**, 395 (2018).
20. Evans D.F., Upton M.W. Studies on singlet oxygen in aqueous solution. Part 4. The 'spontaneous' and catalysed decomposition of hydrogen peroxide. *Dalton Trans.* **12**, 2525-2529 (1985).
21. Koppenol, W.H. Reactions involving singlet oxygen and the superoxide anion. *Nature* **262**, 420-421 (1976).
